# Phytochemical Analysis of the Fruits of Sea Buckthorn (*Hippophae rhamnoides*): Identification of Organic Acid Derivatives

**DOI:** 10.3390/plants10050860

**Published:** 2021-04-24

**Authors:** Yong Hoon Lee, Hee Joo Jang, Kun Hee Park, Seon-Hee Kim, Jung Kyu Kim, Jin-Chul Kim, Tae Su Jang, Ki Hyun Kim

**Affiliations:** 1 School of Pharmacy, Sungkyunkwan University, Suwon 16419, Korea; yhl2090@naver.com (Y.H.L.); dhn01200@naver.com (H.J.J.); 2 Department of Food Science and Biotechnology, Sungkyunkwan University, Suwon 16419, Korea; soske@skku.edu; 3 Sungkyun Biotech Co., Ltd., Suwon 16419, Korea; seonhee31@gmail.com; 4 School of Chemical Engineering, Sungkyunkwan University, Suwon 16419, Korea; legkim@skku.edu; 5 Natural Product Informatics Research Center, KIST Gangneung Institute of Natural Products, Gangneung 25451, Korea; jckim@kist.re.kr; 6 College of Medicine, Dankook University, Cheonan 31116, Korea

**Keywords:** *Hippophae rhamnoides*, Elaeagnaceae, citrate derivatives, nuclear magnetic resonance (NMR), electronic circular dichroism (ECD)

## Abstract

*Hippophae rhamnoides* L. (Elaeagnaceae), commonly known as “Sea buckthorn” and “Vitamin tree”, is a spiny deciduous shrub whose fruit is known for its nutritional composition, such as vitamin C, and is consumed as a dietary supplement worldwide. As part of our ongoing efforts to identify structurally new and bioactive constituents from natural resources, the phytochemical investigation of the extract of *H. rhamnoides* fruits led to the isolation of one malate derivative (**1**), five citrate derivatives (**2–6**), and one quinate derivative (**7**). The structures of the isolated compounds were elucidated by analysis of 1D and 2D nuclear magnetic resonance (NMR) spectroscopic data and high-resolution electrospray ionization (HR-ESI) liquid chromatography–mass spectrometry (LC/MS) data. Three of the citrate derivatives were identified as new compounds: (*S*)-1-butyl-5-methyl citrate (**3**), (*S*)-1-butyl-1′-methyl citrate (**4**), and (*S*)-1-methyl-1′-butyl citrate (**6**), which turned out to be isolation artifacts. The absolute configurations of the new compounds were established by quantum chemical electronic circular dichroism (ECD) calculation, which is an informative tool for verifying the absolute configuration of organic acid derivatives. The isolated compounds **1–7** were evaluated for their stimulatory effects on osteogenesis. Compounds **1**, **3**, **4**, **6**, and **7** stimulated osteogenic differentiation up to 1.4 fold, compared to the negative control. These findings provide experimental evidence that active compounds **1**, **3**, **4**, **6**, and **7** induce the osteogenesis of mesenchymal stem cells and activate bone formation.

## 1. Introduction

*Hippophae rhamnoides* L. (Elaeagnaceae), known as “Sea buckthorn” and “Vitamin tree”, is a spiny deciduous shrub. It has historically been used for medicinal purposes worldwide, especially in Central and Southeastern Asia. In China, its pharmacological use was revealed more than a thousand years ago through the records of Sibu Yidian from the Tang Dynasty and Jing Zhu Ben Cao from the Qing dynasty [1]. Sea buckthorn oil was listed in the Russian Pharmacopeia in the 1950s and used as an anti-inflammatory and wound healing agent in Russia [2]. Its bark and fruits have also been used as Tibetan folk medicine to treat pulmonary disorders, cough, fever, and tumors [3]. For the past 50 years, several medicinal preparations based on *H. rhamnoides* have been clinically used to treat radiation damage, oral inflammation, burns, and gastric ulcers, and more than 300 preparations have been reported in the literature [4].

*H. rhamnoides* bears yellowish or orange berries, which are commonly found as a powdered drink mix in markets as a functional food and are used to make fruit sauce and wine [5]. Vitamin C is a major nutritional component of *H. rhamnoides* fruits [6], the level of which exceeds that of lemons and oranges. Previous pharmacological studies of *H. rhamnoides* have reported that its extracts exhibited therapeutic properties, including anti-platelet effects via the inhibitory mechanism of thrombin-activated platelets and antimicrobial effects against *S. aureus* and *C. albicans* through the inhibition of adhesion and biofilm formation [7,8]. In addition, previous phytochemical investigations on *H. rhamnoides* have demonstrated the presence of diverse types of bioactive substances, including vitamins (A, E, K, riboflavin, and folic acid), carotenoids, phytosterols, minerals, organic acids, polyunsaturated fatty acids, and amino acids [9,10]. Because of the nutritional importance of *H. rhamnoides* fruits, many research groups have attempted to investigate the phytochemical constituents of these fruits, in which flavonoids, proanthocyanidins, alkaloids, and phenolic acids have been identified [11,12]. Nevertheless, previous findings suggested that only few studies have been carried out on the chemical constituents of *H. rhamnoides* fruits, despite their health benefits.

In this context, as a part of continuous research programs to discover structurally new bioactive compounds from diverse natural sources [13,14,15,16,17,18,19], our group has focused on the potential bioactive composition of *H. rhamnoides* fruits. In our recent study of *H. rhamnoides* fruits, we identified six chemical compounds, including one citric acid derivative, two flavonoids, one phenolic compound, and two megastigmane derivatives, which were evaluated for their anti-inflammatory effects by determining LPS-induced NO production in RAW 264.7 cells [20]. As a result, we found that a citric acid derivative, 1,5-dimethyl citrate, effectively inhibited LPS-induced NO production and simultaneously showed anti-inflammatory effects by inhibiting the expression of pro-inflammatory mediators, iNOS and COX-2, and the activity of pro-inflammatory cytokines, IL-6 and TNF-α [20]. Our recent findings provide experimental evidence that the organic acid derivative present in *H. rhamnoides* fruits could function as a promising bioactive substance. In the present study, further phytochemical investigation of the extract of *H. rhamnoides* fruits was performed to identify potential bioactive organic acid constituents based on recent preliminary findings. Phytochemical analysis of the BuOH-soluble fraction derived from the extract led to the isolation of seven organic acid derivatives: one malate derivative (**1**), five citrate derivatives (**2–6**), three of which were identified as new compounds, and one quinate derivative (**7**). The structures of the isolated organic acid derivatives (**1–7**) were determined by analysis of 1D and 2D nuclear magnetic resonance (NMR) spectroscopic data and high-resolution electrospray ionization (HR-ESI) liquid chromatography–mass spectrometry (LC/MS). In addition, the absolute configurations of the new compounds were established using quantum chemical electronic circular dichroism (ECD) calculations. Herein, we describe the isolation and structural elucidation of the organic acid derivatives **1–7** and the evaluation of their biological activities for stimulatory effects on osteogenesis. 

## 2. Results and Discussion

### 2.1. Isolation of Compounds

**Figure 1:**
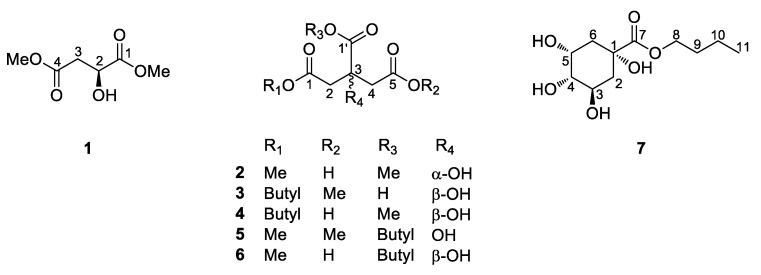
Chemical structure of compounds **1–7**.

The aqueous extract powder of *H. rhamnoides* fruits was suspended for solvent partitioning in water and then fractionated with four organic solvents, namely, hexane, dichloromethane, ethyl acetate, and *n*-butanol. LC/MS-based analysis combined with our in-house built UV library, and TLC analysis of the solvent-partitioned fractions suggested that the BuOH-soluble fraction contained the majority of the organic acid derivatives. Phytochemical analysis of the BuOH-soluble fraction was performed using repeated column chromatography with silica gel 60, RP-C_18_ silica gel, Diaion HP-20, Sephadex LH-20, and high-performance liquid chromatography (HPLC). The final semi-preparative HPLC separation afforded seven organic acid derivatives (**1–7**) (Figure 1). 

### 2.2. Elucidation of Compound Structures

**Figure 2:**
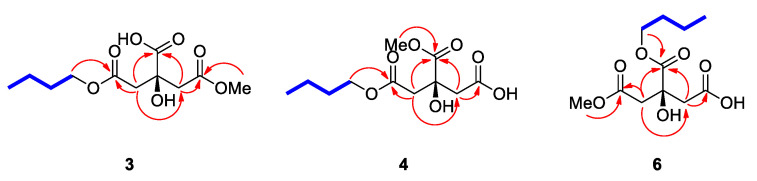
Key ^1^H-^1^H COSY (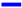) and HMBC (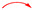) correlations of compounds **3**, **4**, and **6**.

**Figure 3:**
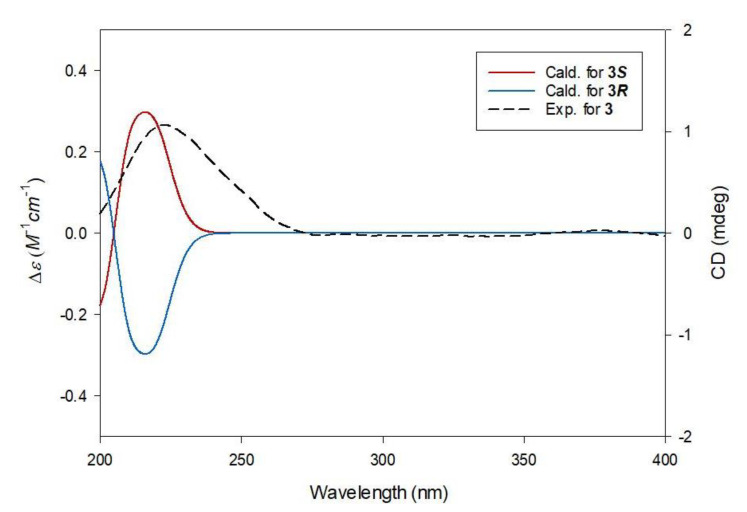
Experimental and calculated ECD spectra of **3**.

Compound **3** was isolated as a yellowish gum. The molecular formula was determined to be C_11_H_18_O_7_ from the protonated molecular ion peak [M + H]^+^ at *m*/*z* 263.1122 (calculated for C_11_H_19_O_7_, 263.1131) in the positive-ion mode of HRESIMS (Figure S1). The ^1^H NMR spectrum (Figure S2) of **3** (Table 1) showed the presence of characteristic signals of an oxygenated methylene group at *δ*_H_ 4.08 (2H, t, *J* = 6.5 Hz), a methoxy group at *δ*_H_ 3.66 (3H, s), two pairs of relatively deshielded methylene groups overlapped at *δ*_H_ 2.84 and 2.92, another two pairs of methylene groups at *δ*_H_ 1.61 (2H, m) and 1.40 (2H, m), and a terminal methyl group at *δ*_H_ 0.94 (3H, t, *J* = 7.5 Hz). The ^13^C NMR data of **3** (Table 1) assigned with the assistance of the HSQC experiment (Figure S4) confirmed the 11 carbon signals, which corresponded to three carboxyl carbons (*δ*_C_ 170.3, 170.5, and 175.3), two oxygenated carbons (*δ*_C_ 64.2 and 72.9), four sets of methylene groups (*δ*_C_ 18.6, 30.2, 42.7, and 42.7), one methoxy-oriented carbon (*δ*_C_ 50.7), and one methyl carbon (*δ*_C_ 12.5). Based on this evidence, it was predicted that compound **3** was a citric acid derivative, since the characteristic NMR data of **3** was similar to that of the citric acid derivatives [21,22]. The gross structure of **3** was determined by 2D NMR experiments (^1^H-^1^H COSY; Figure S3 and HMBC; Figure S5). The HMBC correlations of H_2_-2/C-1, H_2_-2/C-1′, H_2_-2/C-4, H_2_-4/C-1′, H_2_-4/C-2, and H_2_-4/C-5 verified the citric acid moiety. Furthermore, the HMBC correlation of 5-OCH_3_ with C-5 confirmed that the methoxy group was attached at C-5 of the citric acid moiety, and that of H_2_-1″ with C-1, along with ^1^H-^1^H cross-peaks between H_2_-1″/ H_2_-2″/ H_2_-3″/ H_3_-4″, revealed that a butyl group was attached at the other end of C-1 (Figure 2). Thus, the planar structure of **3** was established, as shown in Figure 2. Finally, the absolute configuration at C-3 was assigned using quantum chemical ECD calculations [23,24,25]. Two possibilities with absolute configurations of 3*R* and 3*S* were calculated. The weighted calculated ECD spectrum (green line) of **3*S*** (3*S*) agreed well (although not identical) with the experimental curve of **3**, compared to that (red line) of **3*R*** (3*R*) (Figure 3), thus determining the absolute configuration of C-3 as *S*. The chemical structure of **3**, including its absolute configuration, was elucidated as (*S*)-1-butyl-5-methyl citrate. 

**Figure 4:**
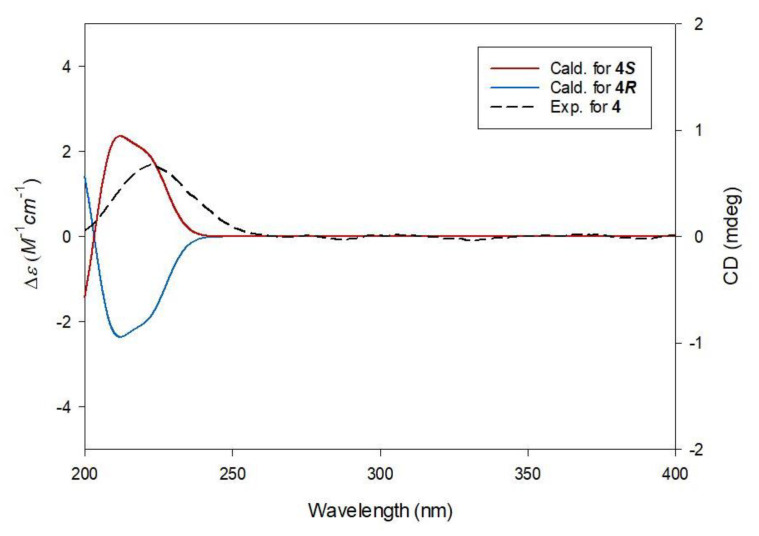
Experimental and calculated ECD spectra of **4**.

Compound **4** was obtained as a white amorphous powder. The molecular formula was determined to be the same as that of compound **3**, C_11_H_18_O_7_, from HRESIMS data (Figure S6), which showed a protonated molecular ion peak [M + H]^+^ at *m*/*z* 263.1119 (calculated for C_11_H_19_O_7_, 263.1131). The ^1^H (Figure S7) and ^13^C NMR data (Figure S9) of **4** (Table 1) were also almost identical to those of compound **3**, except for apparent differences in the chemical shifts responsible for the citric acid moiety—the ^1^H chemical shift of the methoxy group (*δ*_H_ 3.66 in **3**, *δ*_H_ 3.75 in **4**). Detailed analysis of the 2D NMR spectra (^1^H-^1^H COSY; Figure S8 and HMBC; Figure S10) assigned the gross planar structure of **4** (Figure 2), where the key HMBC correlation of 1′-OCH_3_ with C-1′ was observed, indicating that the methoxy group was attached at C-1′ in compound **4** (Figure 2). Finally, to confirm the absolute configuration of C-3 in **4**, the computationally calculated ECD data of the two enantiomers, 3*R-* and 3*S*-isomers, were compared with the experimental ECD data of **4** (Figure 4). The ECD data of **4** showed a good fit with the calculated ECD spectrum (green line) of **4*S*** (3*S*) (Figure 4). This finding allowed us to assign the structure of **4** as (*S*)-1-butyl-1′-methyl citrate.

**Figure 5:**
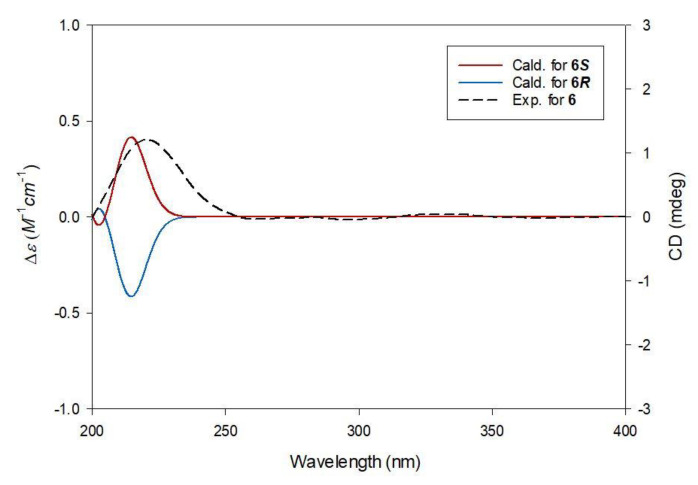
Experimental and calculated ECD spectra of **6**.

Compound **6**, obtained as a white amorphous powder, was determined to have the molecular formula C_11_H_18_O_7_, similar to those of compounds **3** and **4**, from the protonated molecular ion peak [M + H]^+^ at *m*/*z* 263.1145 (calculated for C_11_H_19_O_7_, 263.1131) in the positive-ion mode of HRESIMS (Figure S11). The ^1^H (Figure S12) and ^13^C NMR data (Figure S14) (Table 1) of **6** were also quite similar to those of compounds **3** and **4**, with the only noticeable differences being that the ^13^C chemical shift of C-1 was deshielded to *δ*_C_ 173.9, compared to these two compounds (*δ*_C_ 170.2 in **3**, *δ*_C_ 170.0 in **4**), and the ^1^H chemical shift of H-1″ was deshielded to *δ*_H_ 4.18, compared to **3** and **4** (*δ*_H_ 4.08 in **3**, *δ*_H_ 4.07 in **4**). This finding suggested the possibility that the butyl group in **6** was attached to the different carboxyl groups of compounds **3** and **4**. The gross planar structure of **6** was verified by 2D NMR analysis (^1^H-^1^H COSY; Figure S13 and HMBC; Figure S15) (Figure 2). The key HMBC correlation between 1-OCH_3_ and C-1 supported the presence of the methoxy group at C-1, and the location of the butyl group was determined to be C-1′ by the key HMBC correlation of H-1″ and C-1′ (Figure 2). Finally, the absolute configuration of **6** was assigned using quantum chemical ECD calculations. As shown in Figure 5, the experimental ECD curve of **6** is consistent with the calculated ECD spectrum (green line) of **6*S*** (3*S*). Therefore, the chemical structure of **6** was characterized as (*S*)-1-methyl-1′-butyl citrate. 

The structures of the known compounds (Figure 1) were determined to be (*S*)-dimethyl malate (**1**) [26], (*S*)-5,1′-dimethyl citrate (**2**) [21], 1′-butyl-1,5-dimethyl citrate (**5**) [22], and butyl quinate (**7**) [27] by spectroscopic methods, including ^1^H and ^13^C NMR spectra, by comparing their spectroscopic data with those previously reported in the literature and MS data obtained from LC/MS analysis. To the best of our knowledge, compounds **2**, **5**, and **7** are reported from *H. rhamnoides* for the first time in this study. 

In the meantime, it appeared likely that compounds **3–7**, including the new compounds, arose from the addition of a butyl group to the corresponding carboxylic acid during the experimental procedure, because *n*-BuOH was used as the organic solvent for solvent partitioning. In order to verify whether compounds **3–7** were genuine natural compounds or artifacts, the aqueous extract of *H. rhamnoides* fruits and fractions derived from the extract were subjected to LC/MS analysis, both alone and co-injected with each compound. As a result, there was no peak with molecular ions corresponding to compounds **3–7** in the crude aqueous extract, whereas all the compounds were detected in the BuOH-soluble fraction, suggesting that the compounds with the butyl group may be artifacts produced by the addition of a butyl group during solvent partition with *n*-BuOH. 

### 2.3. Evaluation of Biological Activities of Compounds **1–7**

**Figure 6:**
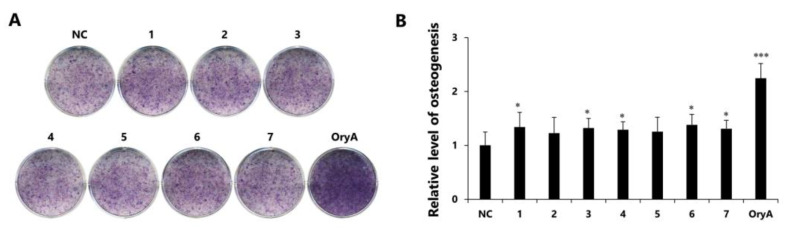
The effects of compounds **1–7** on the differentiation of mesenchymal stem cells (MSCs) toward osteoblasts. (**A**) Stimulatory effect of compounds **1–7** on osteogenic differentiation of MSC. Fully-differentiated C3H10T1/2 cells were stained with alkaline phosphatase (ALP) at 9 d after osteogenic differentiation with 20 µM of compounds **1–7**. (**B**) ALP enzyme activity was measured in osteogenically differentiated C3H10T1/2 cells treated with compounds **1–7**. The values were calculated relatively by setting the untreated negative control to 1. Oryzativol A (5 μM) was added to the experimental set as a positive control and marked as OryA. * denotes *p* < 0.05 and *** denotes *p* < 0.001.

Mesenchymal stem cells (MSCs) in the bone marrow are pluripotent cells that are known to differentiate into osteocytes as well as adipocytes. Since microenvironmental changes cause alterations in the regulation of gene expression in MSC differentiation, where alterations in the expression of the related genes might disturb the balance between osteoprogenitor and adipocyte progenitor cells in osteoporosis patients [28,29,30], a therapy that is able to adjust the gene expression in MSCs would be promising in the management of postmenopausal osteoporosis. To determine the therapeutic effects of the isolated compounds **1–7** in promoting osteogenesis, all the compounds were examined for their effects on the differentiation of murine MSCs into osteoblasts. The murine mesenchymal stem cell line C3H10T1/2 was treated with 20 µM of the compounds during osteogenesis, and the differentiated cells were stained to indicate alkaline phosphatase (ALP) production, which is considered a distinctive marker of osteoblast differentiation [31]. As a result, compounds **1–7** slightly stimulated differentiation of mesenchymal stem cells into osteocytes (Figure 6). Although all the compounds did not show the superior reaction of ALP staining as in the oryzativol A-treated one, the positive control group with compounds **1–7** showed more than 1.2 times the bone differentiation-promoting effects compared to the negative control group. In particular, compounds **1**, **3**, **4**, **6**, and **7** clearly stimulated the differentiation of osteogenic premature cells up to 1.4 times compared to the negative control group (Figure 6).

## 3. Materials and Methods

### 3.1. General Experimental Procedures

Optical rotations were measured using a JASCO P-2000 polarimeter (JASCO, Easton, MD, USA). Ultraviolet (UV) spectra were acquired on an Agilent 8453 UV-visible spectrophotometer (Agilent Technologies, Santa Clara, CA, USA). Electronic circular dichroism (ECD) spectra were measured using a Jasco J-1500 spectropolarimeter (Jasco). Nuclear magnetic resonance (NMR) spectra were recorded using a Bruker AVANCE III (Bruker). HRESIMS spectra were recorded on an Agilent 1290 Infinity II series with a 6545 LC/Q-TOF mass spectrometer (Agilent Technologies). Medium-pressure liquid chromatography (MPLC) was performed on a Smart Flash AKROS (Yamazen, Osaka, Japan) using an analytical Universal ODS-SM 120 Å column (3.0 × 20.0 cm, 50 μm) (Yamazen) and Universal Premium Silica gel 60 Å column (2.3 × 12.3 cm, 30 μm) (Yamazen). Preparative and semi-preparative HPLC were performed on a Waters 1525 binary HPLC pump with a Waters 996 photodiode array detector (Waters Corporation, Milford, CT, USA) using an analytical Agilent Eclipse XDB-C18 column (250 × 21.2 mm, 7 μm) and a Phenomenex Luna Phenyl-hexyl 100 Å column (250 × 10 mm, 10 μm), respectively. LC/MS analysis was performed on an Agilent 1200 series HPLC system with a diode array detector and a 6130 Series ESI mass spectrometer using an analytical Kinetex C_18_ 100 Å column (100 mm × 2.1 mm i.d., 5 μm; flow rate: 0.3 mL/min) (Phenomenex). All HRESIMS data were obtained using an Agilent G6545B quadrupole time-of-flight (Q-TOF) mass spectrometer (Agilent Technologies) with an Agilent EclipsePlus C_18_ column (2.1 mm × 50 mm i.d., 1.8 μm; flow rate: 0.3 mL/min) maintained at 20 °C. Silica gel 60 (230–400 mesh; Merck, Darmstadt, Germany), RP-C_18_ silica gel (Merck, 230–400 mesh), silica Sep-Pak Vac 6 cc cartridges (Waters), and Diaion HP-20 (Mitsubishi Chemical Co. Ltd., Tokyo, Japan) were used for column chromatography. Sephadex LH-20 (Pharmacia, Uppsala, Sweden) was used as the packing material for molecular sieve column chromatography. Thin-layer chromatography (TLC) was performed using precoated silica gel F_254_ plates and RP-C_18_ F_254s_ plates (Merck), and spots were detected under UV light or by heating after spraying with anisaldehyde-sulfuric acid.

### 3.2. Plant Material

Aqueous extract powder of *H. rhamnoides* fruits was purchased from Korea Beauty and Healthcare Co., Ltd. in October 2018. The material was authenticated by one of the authors (K. H. K.). A voucher specimen of the material (VT-2018) was deposited in the herbarium of the School of Pharmacy, Sungkyunkwan University, Suwon, Korea.

### 3.3. Extraction and Isolation

Aqueous extract powder (270 g) of *H. rhamnoides* fruits was suspended in distilled water (700 mL) and then sequentially partitioned with hexane (HX), dichloromethane (MC), ethyl acetate (EA), and *n*-butanol (BuOH). Four layers with different polarities were obtained: HX-soluble (2.7 g), MC-soluble (3.4 g), EA-soluble (7.8 g), and BuOH-soluble (25.2 g) fractions. The BuOH-soluble fraction (25.2 g) was subjected to open-column chromatography on a Diaion HP-20 column and eluted stepwise with distilled water (3 L) and 100% methanol (MeOH). The respective fractions were evaporated to dryness. The resulting MeOH fraction (9.7 g) was chromatographed on silica gel with a gradient solvent system of MC/MeOH (10:1 → 1:1), yielding five subfractions (A–E). Subfraction B (1.2 g) was subjected to an RP-C_18_ column with a gradient solvent system of MeOH/H_2_O (15:85 → 100:0), which afforded six subfractions (B1–B6). Subfraction B2 (393.3 mg) was separated by MPLC on a Yamazen Universal Premium silica gel column with MC/MeOH (99:1 → 95:5) to obtain two subfractions (B21 and B22). Subfraction B21 (219.0 mg) was purified by preparative HPLC (MeOH/H_2_O, 30:70 → 80:20 → 100:0) to obtain four subfractions (B211–B214). Subfraction B212 (58.5 mg) was purified using semi-preparative HPLC (MeCN/H_2_O, 5:95) to furnish compounds **1** (*t*_R_ 29.4 min, 2.2 mg) and **2** (*t*_R_ = 43.2 min, 7.4 mg). Subfraction B4 (356.1 mg) was fractionated by preparative HPLC (MeOH/H_2_O, 70:30 → 100:0) to yield six subfractions (B41–B46). Subfraction B45 (39.3 mg) was purified using semi-preparative HPLC (MeOH/H_2_O, 48:52), and compound **6** (*t*_R_ = 43.1 min, 2.8 mg) was obtained. Subfraction B46 (58.0 mg) was purified using semi-preparative HPLC (MeCN/H_2_O, 25:75), and compounds **4** (*t*_R_ = 35.8 min, 3.1 mg) and **5** (*t*_R_ 71.5 min, 1.8 mg) were obtained. Subfraction C (1.2 g) was fractionated by MPLC using an ODS-SM column with MeOH/ H_2_O (30:70 → 100:0) to yield five subfractions (C1–C5). Subfraction C2 (338.8 mg) was chromatographed using silica Sep-Pak with an isocratic solvent system of MC/MeOH (5:1) to give four subfractions (C21–C24). Subfraction C21 (21.9 mg) was further purified using semi-preparative HPLC (MeOH/H_2_O, 35:65) to yield compound **7** (*t*_R_ = 36.9 min, 7.5 mg). Subfraction D (1.4 g) was separated by preparative HPLC (MeOH/H_2_O, 25:75 → 90:10 → 100:0), and two subfractions were obtained (D1 and D2). Subfraction D2 (446.2 mg) was fractionated by preparative HPLC again with a gradient solvent system of MeOH/H_2_O (70:30 → 85:15), which afforded three subfractions (D21–D23). Subfraction D23 (140.1 mg) was purified using semi-preparative HPLC (MeOH/H_2_O, 40:60 → 50:50) to yield compound **3** (*t*_R_ = 50.8 min, 5.7 mg). 

#### 3.3.1. (*S*)-1-Butyl-5-Methyl Citrate (**3**)

Yellowish gum; ${\left[\alpha \right]}_{D}^{25}$ +6.0 (*c* 0.50, MeOH); UV (MeOH) λ_max_ (log ε) 200 (3.9), 220 (1.5) nm; ECD (MeOH, 0.89% *w*/*v*) λ_max_ (Δε) 217 (+1.58) nm; ^1^H (850 MHz) and ^13^C (212.5 MHz) NMR data, see Table 1; ESIMS (positive-ion mode) *m*/*z* 263.1 [M + H]^+^; HRESIMS (positive-ion mode) *m*/*z* 263.1122 [M + H]^+^ (calcd for C_11_H_19_O_7_, 263.1131).

#### 3.3.2. (*S*)-1-Butyl-1′-Methyl Citrate (**4**)

White amorphous powder; ${\left[\alpha \right]}_{D}^{25}$ −5.4 (*c* 1.65, MeOH); UV (MeOH) λ_max_ (log ε) 201 (3.8), 220 (1.4) nm; ECD (MeOH, 0.94% *w*/*v*) λ_max_ (Δε) 222 (+0.72) nm; ^1^H (850 MHz) and ^13^C (212.5 MHz) NMR data, see Table 1; ESIMS (positive-ion mode) *m*/*z* 263.1 [M + H]^+^; HRESIMS (positive-ion mode) *m*/*z* 263.1119 [M + H]^+^ (calcd for C_11_H_19_O_7_, 263.1131).

#### 3.3.3. (*S*)-1-Methyl-1′-Butyl Citrate (**6**)

White amorphous powder; ${\left[\alpha \right]}_{D}^{25}$ −24.3 (*c* 1.41, MeOH); UV (MeOH) λ_max_ (log ε) 198 (3.9), 218 (1.5) nm; ECD (MeOH, 0.92% *w*/*v*) λ_max_ (Δε) 217 (+1.71) nm; ^1^H (850 MHz) and ^13^C (212.5 MHz) NMR data, see Table 1; ESIMS (positive-ion mode) *m*/*z* 263.1 [M + H]^+^; HRESIMS (positive-ion mode) *m*/*z* 263.1145 [M + H]^+^ (calcd for C_11_H_19_O_7_, 263.1131).

### 3.4. Computational Analysis

All conformers proposed in this study were acquired through the MacroModel (version 2019-3, Schrödinger LLC) module with mixed torsional/low-mode sampling, implemented with the MMFF94 force field. All searches were initially set in the gas phase, with a 10 kJ/mol energy window limit and a maximum of 10,000 steps to thoroughly explore all potential conformers. The Polak–Ribiere conjugate gradient protocol was established with 10,000 maximum iterations and a 0.001 kJ (mol Å)^−1^ convergence threshold on the rms gradient to minimize conformers [23,24,25]. Conformers proposed in this study (within 5 kJ/mol found in the MMFF force field) were selected for geometry optimization by Tmolex 4.3.1, with the DFT settings of B3-LYP/6-31+G(d,p). ECD calculations for **3*R*/3*S*, 4*R*/4*S,*** and **6*R*/6*S*** conformers were performed at identical theoretical levels and basis sets. The calculated ECD spectra were simulated by superimposing each transition, where σ is the width of the band at a height of 1/e. Δ*E*_i_ and *R*_i_ are the excitation energies and rotatory strengths for transition *i*, respectively. In this study, the value of *σ* was 0.10 eV. The excitation energies and rotational strengths for ECD spectra were calculated based on the Boltzmann populations of the conformers, and ECD visualization was carried out using SigmaPlot 14.0.

$$\Delta \epsilon \left(E\right)=\frac{1}{2.297\times 10^{-39}}\frac{1}{\sqrt{2\pi \sigma }}\overset{i}{\underset{A}{\sum }}\Delta E_{i}R_{i}e^{[-{\left(E-\Delta E_{i}\right)}^{2}/\left(2\sigma {)}^{2}\right]}$$

### 3.5. Cell Culture and Differentiation

Mouse mesenchymal stem cell line C3H10T1/2 cells were cultured in Dulbecco’s modified Eagle’s medium (DMEM), 10% fetal bovine serum (FBS), and 1% penicillin-streptomycin (P/S) at 37 °C in a 5% CO₂ incubator. For osteogenic differentiation, C3H10T1/2 cells were exposed to DMEM (5% FBS, 1% P/S) media with 10 mM β-glycerophosphate and 50 μg/mL ascorbic acid for 9 d. During osteogenic differentiation, 20 µM of compounds **1–7** were added to the cells, and 5 µM of oryzativol A was used as the positive control. 

To investigate the degree of differentiation into osteocytes, cells were stained for alkaline phosphatase (ALP) after 9 days of osteogenic differentiation. After fixation with 4% formaldehyde solution, cells were exposed to 0.4 mg/mL of nitroblue tetrazolium and 0.2 mg/mL of 5-bromo-4chloro-3′-indolyphosphate in alkaline phosphatase (AP) buffer (100 mM Tris-HCl, pH 9.5; 100 mM NaCl, 5 mM MgCl_2_) in the dark for 4 h. Next, the reaction was stopped with 5 mM EDTA. To measure APL activity, cell lysates were collected from the differentiated osteogenic cells and treated with *p*-nitrophenyl phosphate (*p*-NPP) solution (Alkaline Phosphatase Assay Kit (ab83369; Abcam, Cambridge, MA, USA). Color changes of the samples were measured at 405 nm with a SpectraMax M2 Microplate Spectrophotometer. 

### 3.6. Statistical Analysis

Each sample was tested in triplicate, and the test was repeated three times. Data are presented as the mean ± standard deviation (S.D.). Differences between the control and experimental groups were analyzed using a two-tailed unpaired Student’s *t*-test*;* statistical significance was defined as *p* < 0.05.

## 4. Conclusions

In this study, phytochemical examination of the extracts of *H. rhamnoides* fruits resulted in the isolation of seven organic acid derivatives: one malate derivative (**1**), five citrate derivatives (**2–6**), and one quinate derivative (**7**). The structure of the isolates was elucidated by analysis of 1D and 2D NMR data and HR-ESIMS data as well as quantum chemical ECD calculations. Three of the citrate derivatives were identified as new compounds: (*S*)-1-butyl-5-methyl citrate (**3**), (*S*)-1-butyl-1′-methyl citrate (**4**), and (*S*)-1-methyl-1′-butyl citrate (**6**), which turned out to be isolation artifacts. To the best of our knowledge, compounds **2**, **5**, and **7** are reported for the first time from *H. rhamnoides* in this study. An osteogenesis-promoting activity bioassay in C3H10T1/2 cells demonstrated that compounds **1–7** showed little more than 1.2 times the bone differentiation promoting effect, compared to the negative control group. In particular, compounds **1**, **3**, **4**, **6**, and **7** clearly stimulated the differentiation of osteogenic premature cells up to 1.4 times, compared to the negative control group. Although the stimulatory effect of the active compounds on osteogenic differentiation was far behind that of the positive control, oryzativol A, compounds **1**, **3**, **4**, **6**, and **7** apparently helped to differentiate the mesenchymal stem cells into osteocytes.

## Figures and Tables

**Table 1:** ^1^H (850 MHz) and ^13^C (212.5 MHz) NMR data of compounds **3**, **4**, and **6** in CD_3_OD ^a^.

Position	3		4		6	
	*δ*_H_	*δ*_C_	*δ*_H_	*δ*_C_	*δ*_H_	*δ*_C_
1	-	170.2	-	170.0	-	170.5
2	2.92 m ^b^ 2.84 m ^b^	42.7 ^b^	2.92 d (15.5) 2.76 d (15.5)	42.2 ^b^	2.93 d (15.5) 2.79 d (15.5)	43.1
3	-	72.9	-	73.2		73.2
4	2.92 m ^b^ 2.84 m ^b^	42.7 ^b^	2.88 d (15.5) 2.72 d (15.5)	42.2 ^b^	2.85 d (15.5) 2.70 d (15.5)	42.8
5	-	170.5	-	172.6	-	173.9 ^b^
1′	-	175.3	-	174.1	-	173.9 ^b^
1′-OCH_3_	-	-	3.75 s	51.5	-	-
1-OCH_3_	-	-	-	-	3.67 s	50.2
5-OCH_3_	3.66 s	50.7	-	-	-	-
1″	4.08 t (6.5)	64.2	4.07 t (6.5)	64.2	4.18 t (6.5)	64.9
2″	1.61 m	30.2	1.61 m	30.2	1.67 m	30.0
3″	1.40 m	18.6	1.39 m	18.6	1.43 m	18.6
4″	0.94 t (7.5)	12.5	0.94 t (7.5)	12.5	0.97 t (7.5)	12.5

^a^ Coupling constants (in Hz) are given in parentheses. Assignments based on ^1^H-^1^H COSY, HSQC, and HMBC. ^b^ Overlapped.

## Supplementary Materials

1D and 2D NMR, and HR-ESIMS of compounds **3**, **4**, and **6** are available online at https://www.mdpi.com/article/10.3390/plants10050860/s1, Figure S1: HR-ESIMS data of **3**, Figure S2: ^1^H NMR spectrum of **3** (CD_3_OD, 700 MHz), Figure S3: ^1^H-^1^H COSY spectrum of **3** (CD_3_OD), Figure S4: HSQC spectrum of **3** (CD_3_OD), Figure S5: HMBC spectrum of **3** (CD_3_OD), Figure S6: HR-ESIMS data of **4**, Figure S7: ^1^H NMR spectrum of **4** (CD_3_OD, 700 MHz), Figure S8: ^1^H-^1^H COSY spectrum of **4** (CD_3_OD), Figure S9: HSQC spectrum of **4** (CD_3_OD), Figure S10: HMBC spectrum of **4** (CD_3_OD), Figure S11: HR-ESIMS data of **6**, Figure S12: ^1^H NMR spectrum of **6** (CD_3_OD, 700 MHz), Figure S13: ^1^H-^1^H COSY spectrum of **6** (CD_3_OD), Figure S14: HSQC spectrum of **6** (CD_3_OD), Figure S15: HMBC spectrum of **6** (CD_3_OD), Table S1: Gibbs free energies and Boltzmann distribution of conformers **3*S*** (3*S*), Table S2: Gibbs free energies and Boltzmann distribution of conformers **4*S*** (3*S*), Table S3: Gibbs free energies and Boltzmann distribution of conformers **6*S*** (3*S*).

## References

[1] Mingyu X., Xiaoxuan S., Jinhua C. (1991). The medicinal research and development of seabuckthorn. J. Water Soil Conserv..

[2] Panossian A., Wagner H. (2013). From traditional to evidence-based use of *Hippophae rhamnoides* L.: Chemical composition, experimental, and clinical pharmacology of sea buckthorn berries and leaves extracts. Evidence and Rational Based Research on Chinese Drugs.

[3] Watanabe T., Rajbhanddari K.R., Malla K.J., Yahara S. (2005). A handbook of medicinal plants of Nepal. Banko Janakari.

[4] Chauhan A.S., Negi P.S., Ramteke R.S. (2007). Antioxidant and antibacterial activities of aqueous extract of Seabuckthorn (*Hippophae rhamnoides*) seeds. Fitoterapia.

[5] Suryakumar G., Gupta A. (2011). Medicinal and therapeutic potential of Sea buckthorn (*Hippophae rhamnoides* L.). J. Ethnopharmacol..

[6] Li T.S., Schroeder W.R. (1996). Sea buckthorn (*Hippophae rhamnoides* L.): A multipurpose plant. HortTechnology.

[7] Skalski B., Kontek B., Rolnik A., Olas B., Stochmal A., Żuchowski J. (2019). Anti-platelet properties of phenolic extracts from the leaves and twigs of *Elaeagnus rhamnoides* (L.) A. Nelson. Molecules.

[8] Różalska B., Sadowska B., Żuchowski J., Więckowska-Szakiel M., Budzyńska A., Wójcik U., Stochmal A. (2018). Phenolic and nonpolar fractions of *Elaeagnus rhamnoides* (L.) A. Nelson, extracts as virulence modulators-in vitro study on bacteria, fungi, and epithelial cells. Molecules.

[9] Beveridge T., Li T.S., Oomah B.D., Smith A. (1999). Sea buckthorn products: Manufacture and composition. J. Agri. Food Chem..

[10] Yang B., Kallio H.P. (2001). Fatty acid composition of lipids in sea buckthorn (*Hippophaë rhamnoides* L.) berries of different origins. J. Agri. Food Chem..

[11] Giuffrida D., Pintea A., Dugo P., Torre G., Pop R.M., Mondello L. (2012). Determination of Carotenoids and their Esters in Fruits of Sea Buckthorn (*Hippophae rhamnoides* L.) by HPLC-DAD-APCI-MS. Phytochem. Anal..

[12] Zheng R.X., Xu X.D., Tian Z., Yang J.S. (2009). Chemical constituents from the fruits of *Hippophae rhamnoides*. Nat. Prod. Res..

[13] Lee S., Lee D., Ryoo R., Kim J.C., Park H.B., Kang K.S., Kim K.H. (2020). Calvatianone, a Sterol Possessing a 6/5/6/5-Fused Ring System with a Contracted Tetrahydrofuran B-Ring, from the Fruiting Bodies of *Calvatia nipponica*. J. Nat. Prod..

[14] Lee S.R., Kang H.S., Yoo M.J., Yi S.A., Beemelmanns C., Lee J.C., Kim K.H. (2020). Anti-adipogenic Pregnane Steroid from a Hydractinia-associated Fungus, *Cladosporium sphaerospermum* SW67. Nat. Prod. Sci..

[15] Lee S., Ryoo R., Choi J.H., Kim J.H., Kim S.H., Kim K.H. (2020). Trichothecene and tremulane sesquiterpenes from a hallucinogenic mushroom *Gymnopilus junonius* and their cytotoxicity. Arch. Pharm. Res..

[16] Trinh T.A., Park E.J., Lee D., Song J.H., Lee H.L., Kim K.H., Kim Y., Jung K., Kang K.S., Yoo J.E. (2019). Estrogenic activity of sanguiin H-6 through activation of estrogen receptor α coactivator-binding site. Nat. Prod. Sci..

[17] Ha J.W., Kim J., Kim H., Jang W., Kim K.H. (2020). Mushrooms: An Important Source of Natural Bioactive Compounds. Nat. Prod. Sci..

[18] Yu J.S., Li C., Kwon M., Oh T., Lee T.H., Kim D.H., Ahn J.S., Ko S.K., Kim C.S., Cao S. (2020). Herqueilenone a, a unique rearranged benzoquinone-chromanone from the hawaiian volcanic soil-associated fungal strain *Penicillium herquei* FT729. Bioorg. Chem..

[19] Yu J.S., Park M., Pang C., Rashan L., Jung W.H., Kim K.H. (2020). Antifungal phenols from *Woodfordia uniflora* collected in Oman. J. Nat. Prod..

[20] Baek S.C., Lee D., Jo M.S., Lee K.H., Lee Y.H., Kang K.S., Yamabe N., Kim K.H. (2020). Inhibitory effect of 1,5-dimethyl citrate from sea buckthorn (*Hippophae rhamnoides*) on lipopolysaccharide-induced inflammatory response in RAW 264.7 Mouse Macrophages. Foods.

[21] Takeuchi Y., Nagao Y., Toma K., Yoshikawa Y., Akiyama T., Nishioka H., Abe H., Harayama T., Yamamoto S. (1999). Synthesis and siderophore activity of vibrioferrin and one of its diastereomeric isomers. Chem. Pharm. Bull..

[22] Vereshchagin A.L., Anikina E.V., Syrchina A.I., Lapin M.F., Azin L.A., Semenov A.A. (1989). Chemical investigation of the bitter substances of the fruit of *Lonicera caerulea*. Chem. Nat. Compd.

[23] Lee S.R., Seok S., Ryoo R., Choi S.U., Kim K.H. (2019). Macrocyclic trichothecene mycotoxins from a deadly poisonous mushroom, *Podostroma cornu-damae*. J. Nat. Prod..

[24] Baek S.C., Lee B.S., Yi S.A., Yu J.S., Lee J., Ko Y.J., Pang C., Kim K.H. (2020). Discovery of dihydrophaseic acid glucosides from the florets of *Carthamus tinctorius*. Plants.

[25] Rischer M., Lee S.R., Eom H.J., Park H.B., Vollmers J., Kaster A.K., Shin Y.H., Oh D.C., Kim K.H., Beemelmanns C. (2019). Spirocyclic cladosporicin A and cladosporiumins I and J from a *Hydractinia*-associated *Cladosporium sphaerospermum* SW67. Org. Chem. Front..

[26] Jin T.Y., Shen T., Zhou M.X., Li A.L., Feng D., Zheng B., Gong J., Sun J., Li L., Xiang L. (2016). Chemical constituents from *Portulaca oleracea* and their bioactivities. J. Chin. Pharm. Sci..

[27] Yang Y.B., Li X., Yang Q., Wu Z.J., Sun L.N. (2009). Study on chemical constituents of *Papaya rugosa*. Acad. J. Second Mil. Medi. Univ..

[28] Meyer M.B., Benkusky N.A., Sen B., Rubin J., Pike J.W. (2016). Epigenetic Plasticity Drives Adipogenic and Osteogenic Differentiation of Marrow derived Mesenchymal Stem Cells. J. Biol. Chem..

[29] Ciuffreda M.C., Malpasso G., Musarò P., Turco V., Gnecchi M. (2016). Protocols for in vitro Differentiation of Human Mesenchymal Stem Cells into Osteogenic, Chondrogenic and Adipogenic Lineages. Methods Mol. Biol..

[30] Yi S.A., Lee J., Park S.K., Kim J.Y., Park J.W., Lee M.G., Nam K.H., Park J.H., Oh H., Kim S. (2020). Fermented ginseng extract, BST204, disturbs adipogenesis of mesenchymal stem cells through inhibition of S6 kinase 1 signaling. J. Ginseng Res..

[31] Kang M.H., Lee S.J., Lee M.H. (2020). Bone remodeling effects of Korean Red Ginseng extracts for dental implant applications. J. Ginseng Res..

