# Supplementary material for: Phytochemical Analysis of the Fruits of Sea Buckthorn (Hippophae rhamnoides): Identification of Organic Acid Derivatives
Source: Plants (Basel). 2021 Apr 24;10(5):860. doi: 10.3390/plants10050860 (PMC8146194; doi:10.3390/plants10050860)
Supplement: Supplementary file 1 [file plants-10-00860-s001.zip › plants-1192411-supplementary.pdf]

Supplementary Material

Phytochemical Analysis of the Fruits of Sea Buckthorn (*Hippophae rhamnoides*): Identification of Organic Acid Derivatives

Yong Hoon Lee [1,†], Hee Joo Jang [1,†], Kun Hee Park [2], Seon-Hee Kim [3], Jung Kyu Kim [4], Jin-Chul Kim [5], Tae Su Jang [6,*], and Ki Hyun Kim [1,*]

[1] School of Pharmacy, Sungkyunkwan University, Suwon 16419, Republic of Korea; yhl2090@naver.com (Y.H.L.); dhn01200@naver.com (H.J.J.)
[2] Department of Food Science and Biotechnology, Sungkyunkwan University, Suwon 16419; Republic of Korea; soske@skku.edu (K.H.P.)
[3] Sungkyun Biotech Co., Ltd., Suwon, Gyeonggi-do, 16419, Republic of Korea; seonhee31@gmail.com (S.H.K.)
[4] School of Chemical Engineering, Sungkyunkwan University, Suwon, Gyeonggi-do, 16419, Republic of Korea; legkim@skku.edu (J.K.K.)
[5] KIST Gangneung Institute of Natural products, Natural Product Informatics Research Center, Gangneung, 25451, Republic of Korea; jckim@kist.re.kr (J.C.K.)
[6] College of Medicine, Dankook University, Cheonan 31116, Republic of Korea
* Correspondence: jangts@dankook.ac.kr (T.S.J.); khkim83@skku.edu (K.H.K.); Tel.: +82-41-550-1476 (T.S.J.); +82-31-290-7700 (K.H.K.)
† These authors contributed equally to this study.

Supporting Information Contents:

Figure S1. HR-ESIMS data of **3**

Figure S2. ^{1}H NMR spectrum of **3** (CD_3OD, 700 MHz)

Figure S3. ^{1}H-^{1}H COSY spectrum of **3** (CD_3OD)

Figure S4. HSQC spectrum of **3** (CD_3OD)

Figure S5. HMBC spectrum of **3** (CD_3OD)

Figure S6. HR-ESIMS data of **4**

Figure S7. ^{1}H NMR spectrum of **4** (CD_3OD, 700 MHz)

Figure S8. ^{1}H-^{1}H COSY spectrum of **4** (CD_3OD)

Figure S9. HSQC spectrum of **4** (CD_3OD)

Figure S10. HMBC spectrum of **4** (CD_3OD)

Figure S11. HR-ESIMS data of **6**

Figure S12. ^{1}H NMR spectrum of **6** (CD_3OD, 700 MHz)

Figure S13. ^{1}H-^{1}H COSY spectrum of **6** (CD_3OD)

Figure S14. HSQC spectrum of **6** (CD_3OD)

Figure S15. HMBC spectrum of **6** (CD_3OD)

Table S1. Gibbs free energies and Boltzmann distribution of conformers **3*S*** (3*S*)

Table S2. Gibbs free energies and Boltzmann distribution of conformers **4*S*** (3*S*)

Table S3. Gibbs free energies and Boltzmann distribution of conformers **6*S*** (3*S*)

Cartesian coordinates of conformers of 1 for Tables S1-S3

Figure S1. HR-ESIMS data of **3**

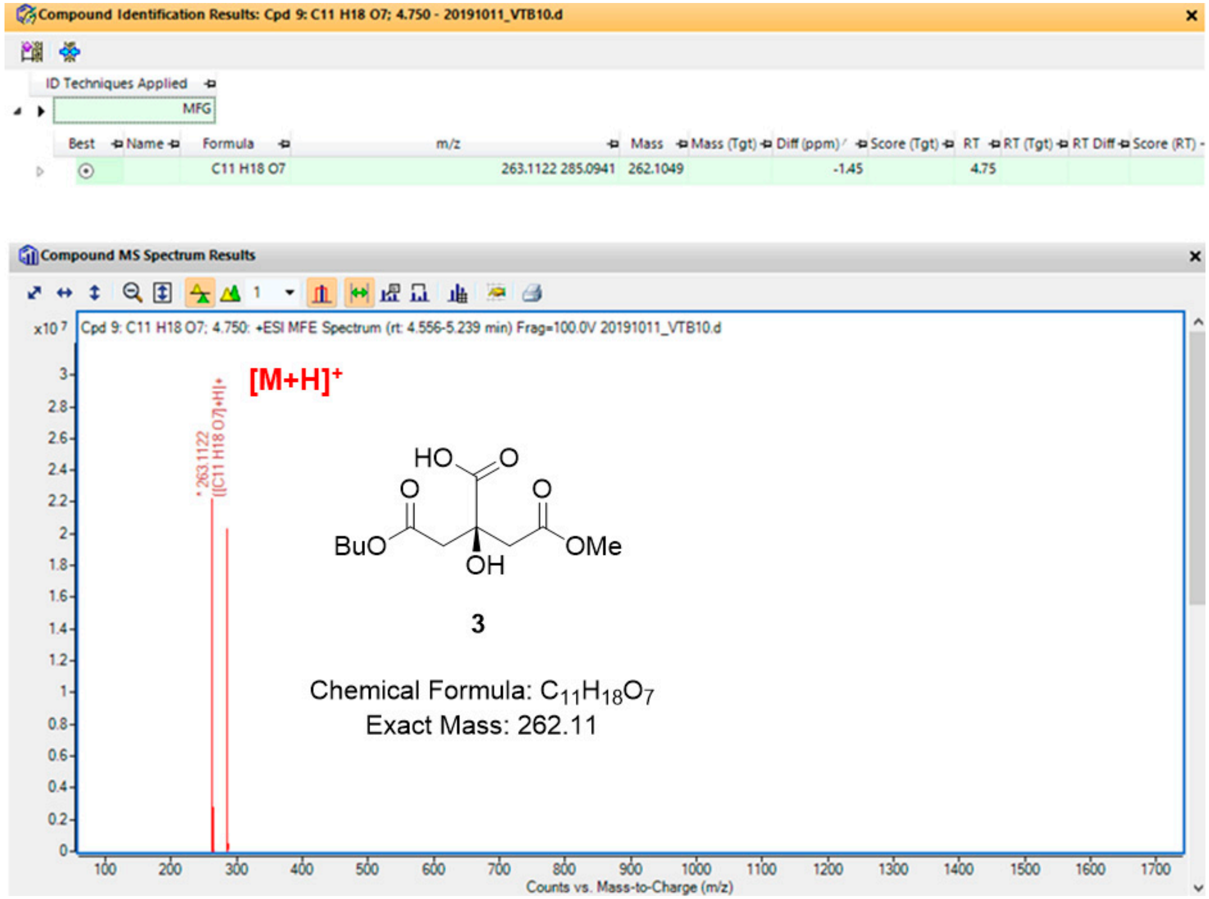

Figure S2. ^{1}H NMR spectrum of **3** (CD_3OD, 700 MHz)

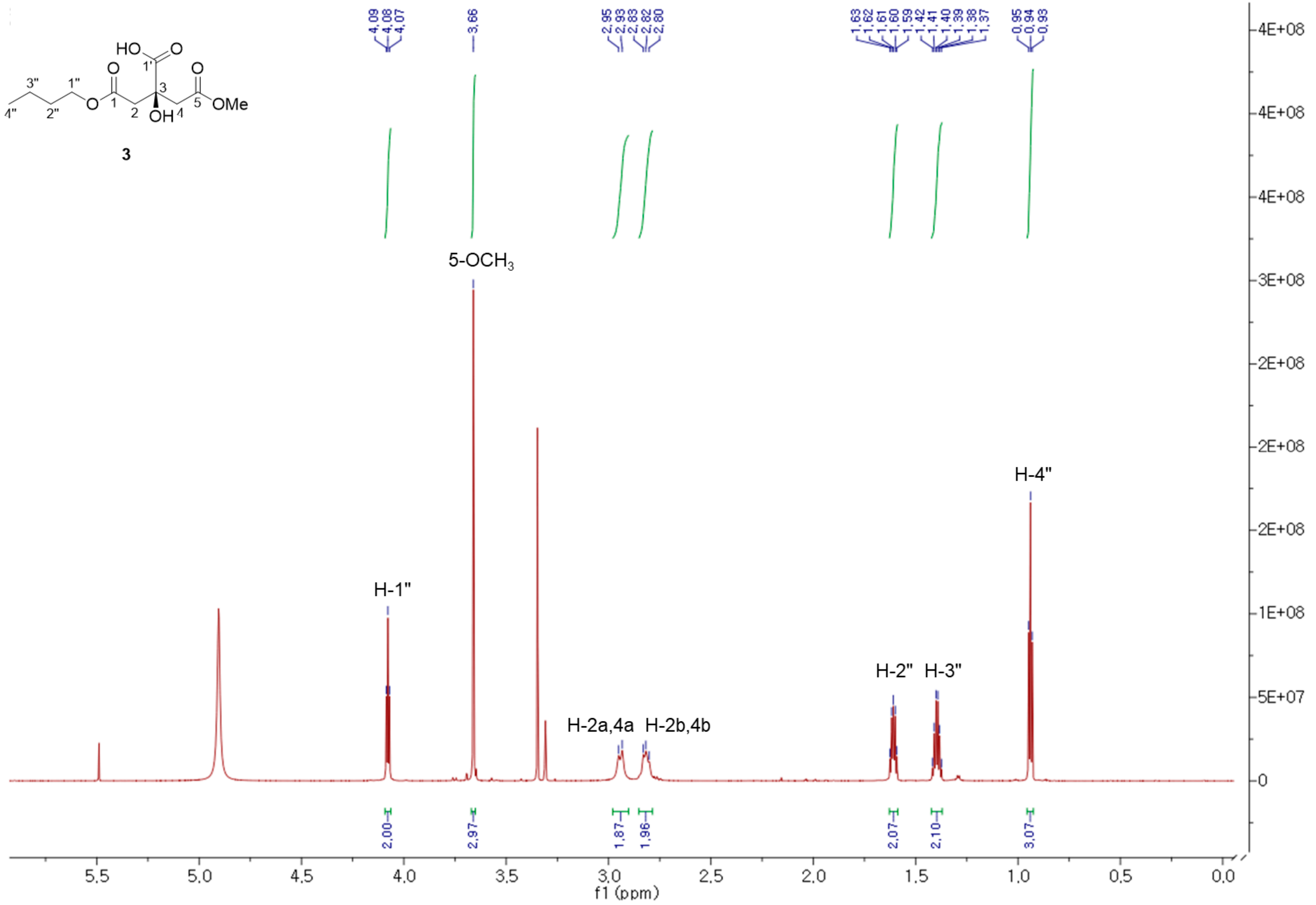

Figure S3. ^{1}H-^{1}H COSY spectrum of **3** (CD_3OD)

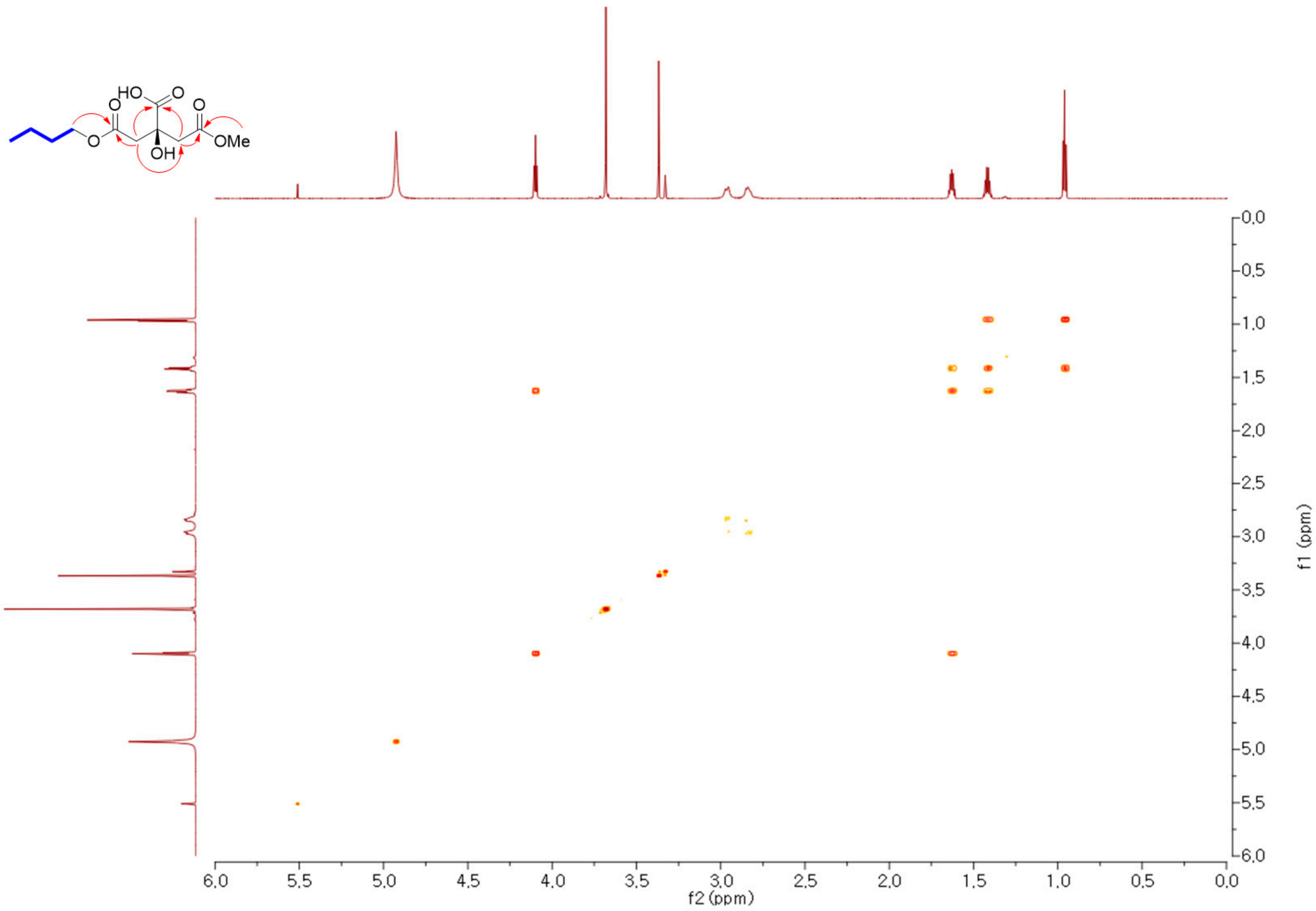

Figure S4. HSQC spectrum of **3** (CD_3OD)

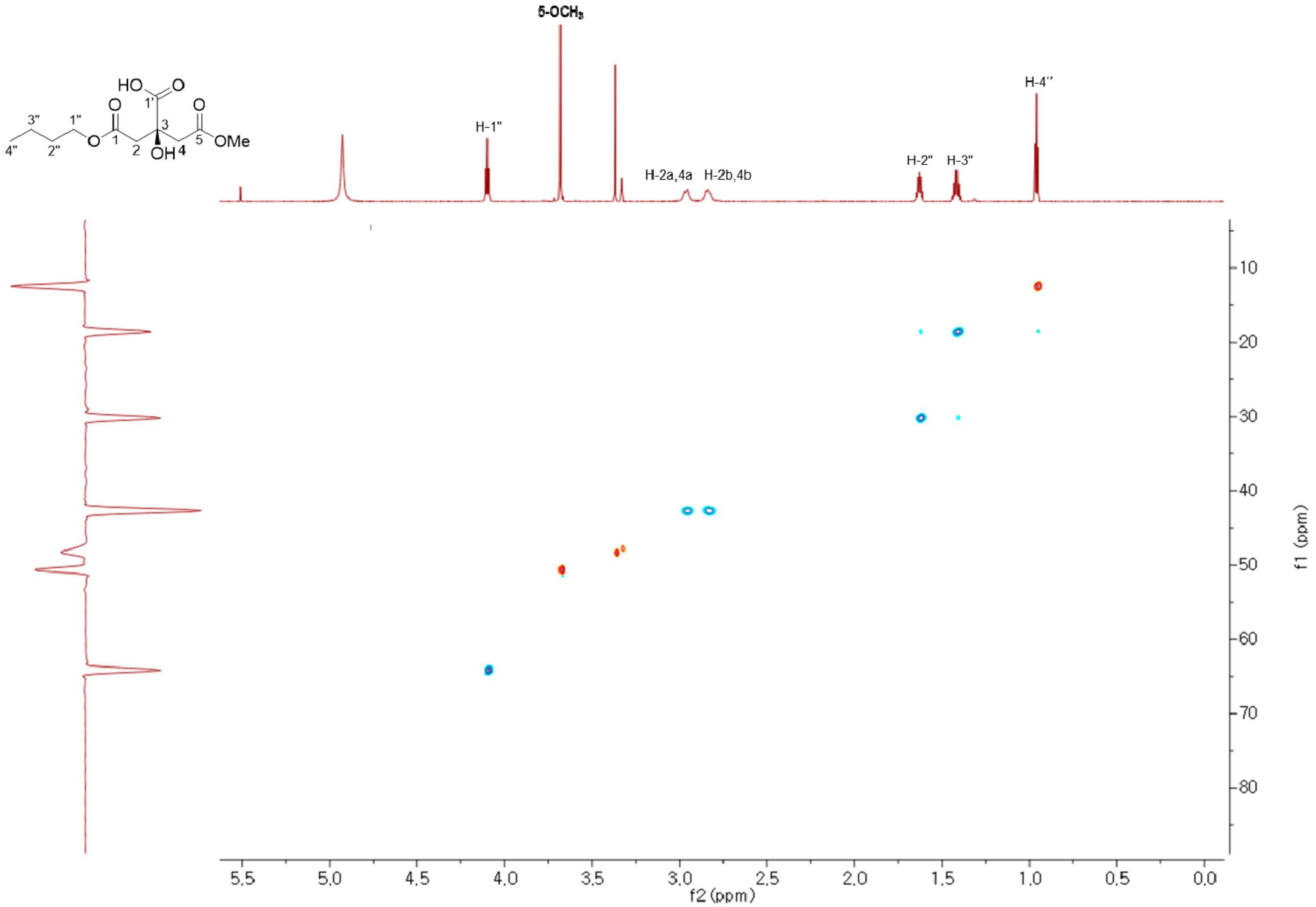

Figure S5. HMBC spectrum of **3** (CD_3OD)

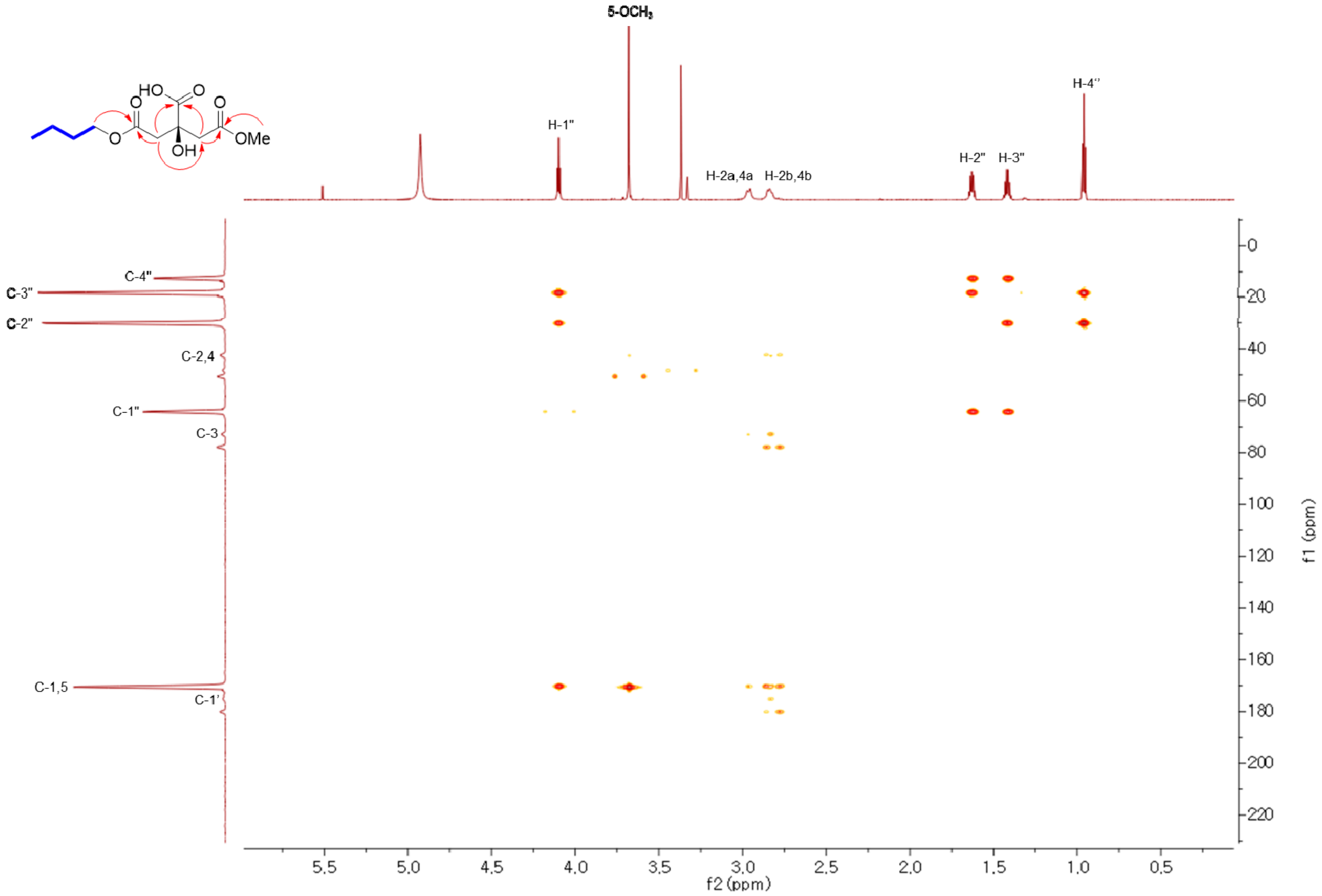

Figure S6. HR-ESIMS data of **4**

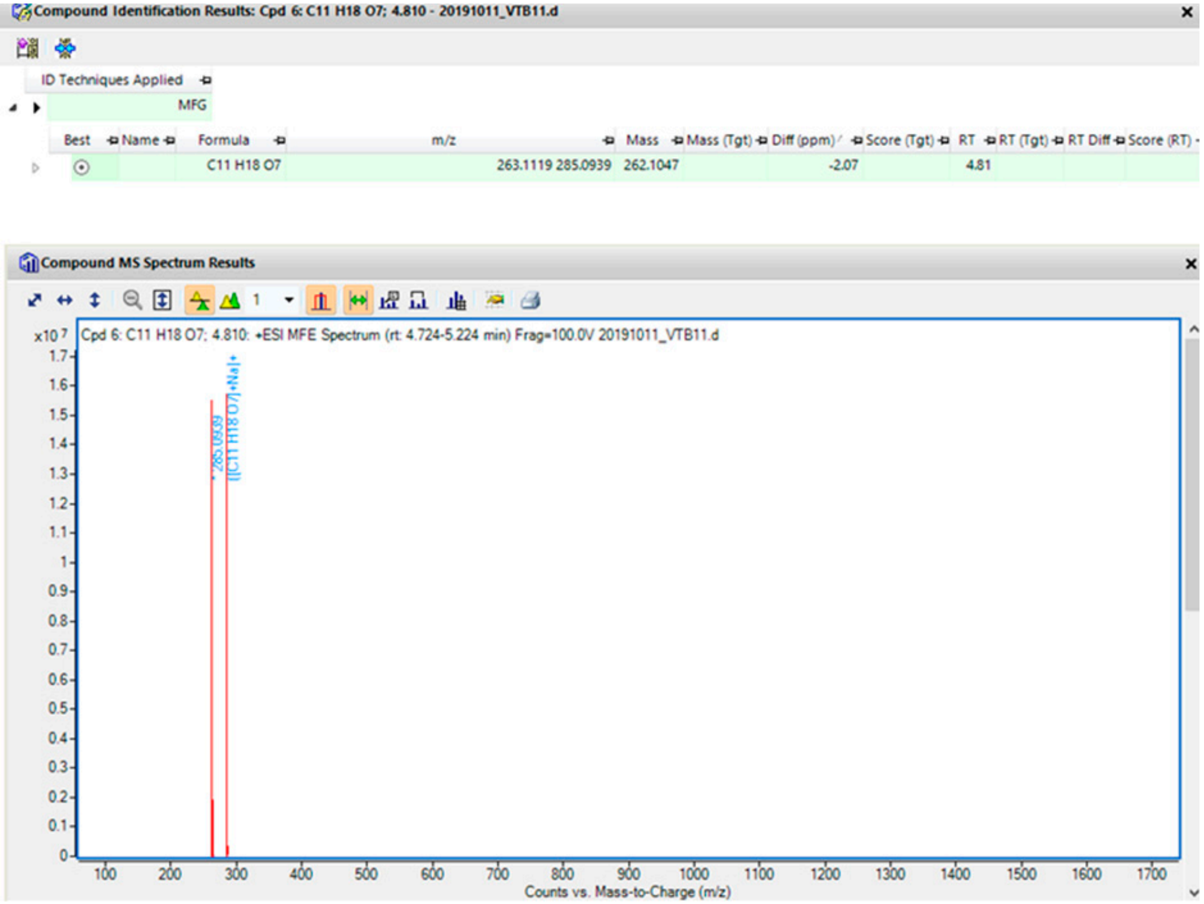

Figure S7. ^{1}H NMR spectrum of **4** (CD_3OD, 700 MHz)

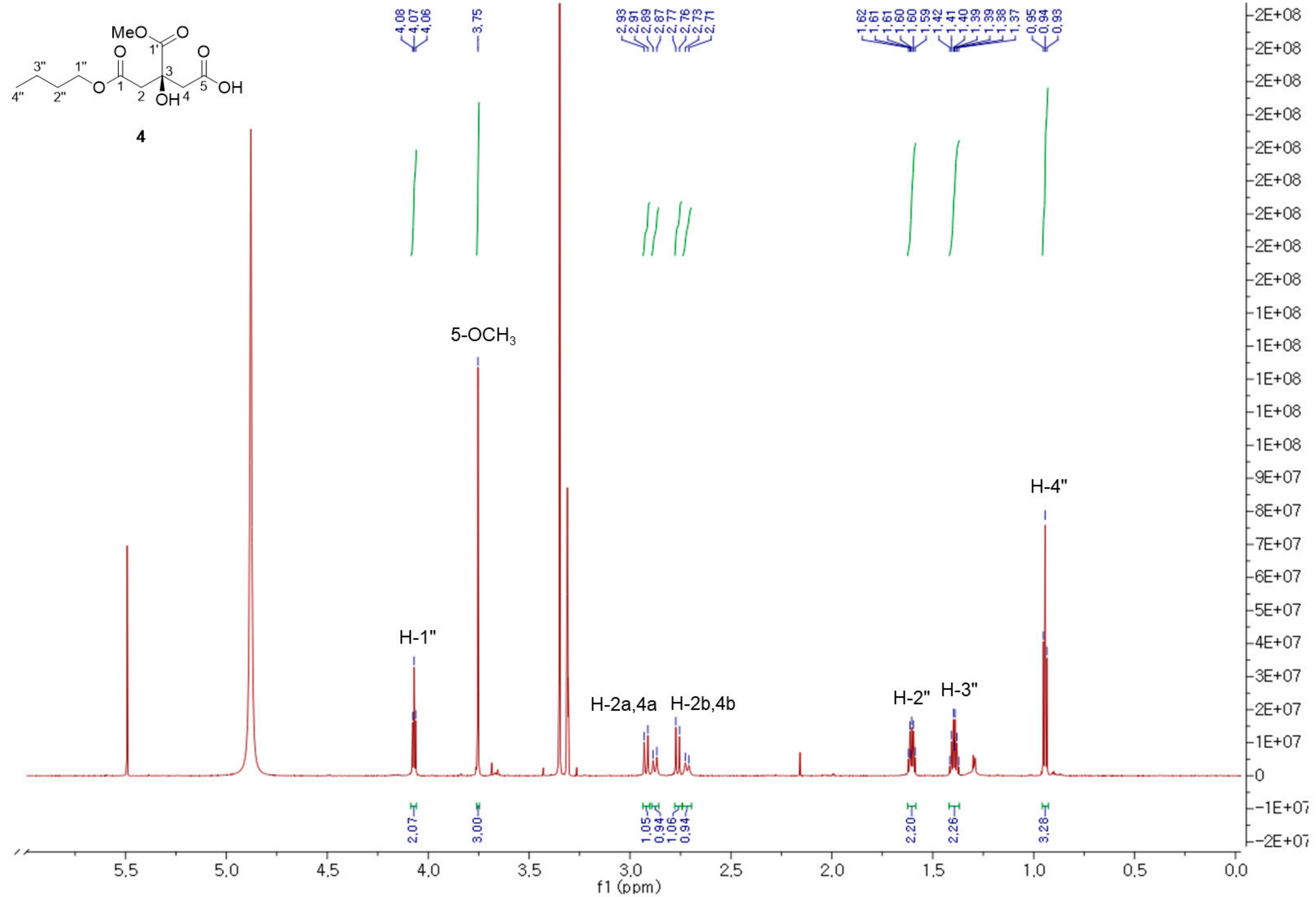

Figure S8. ^{1}H-^{1}H COSY spectrum of **4** (CD_3OD)

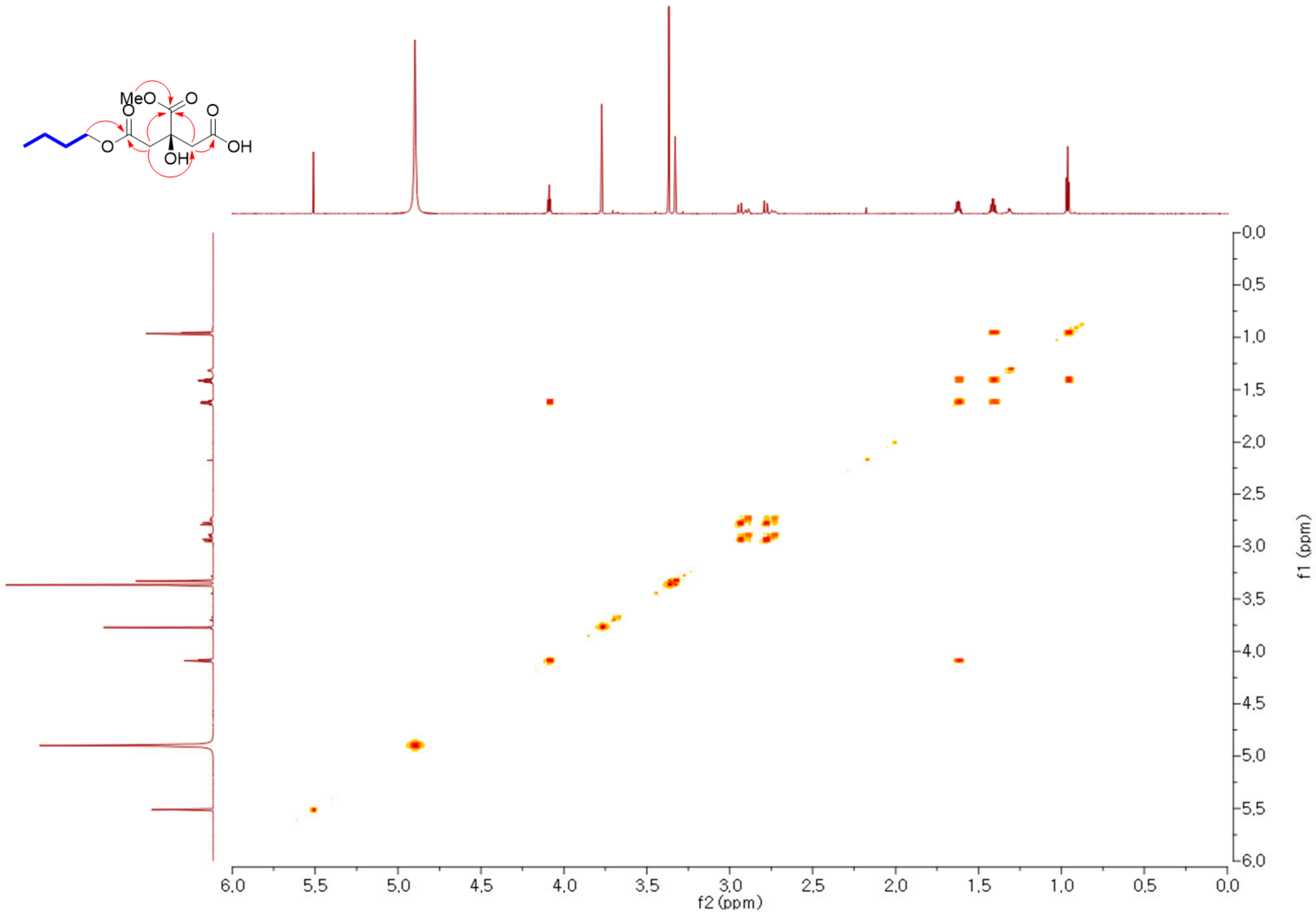

Figure S9. HSQC spectrum of **4** (CD_3OD)

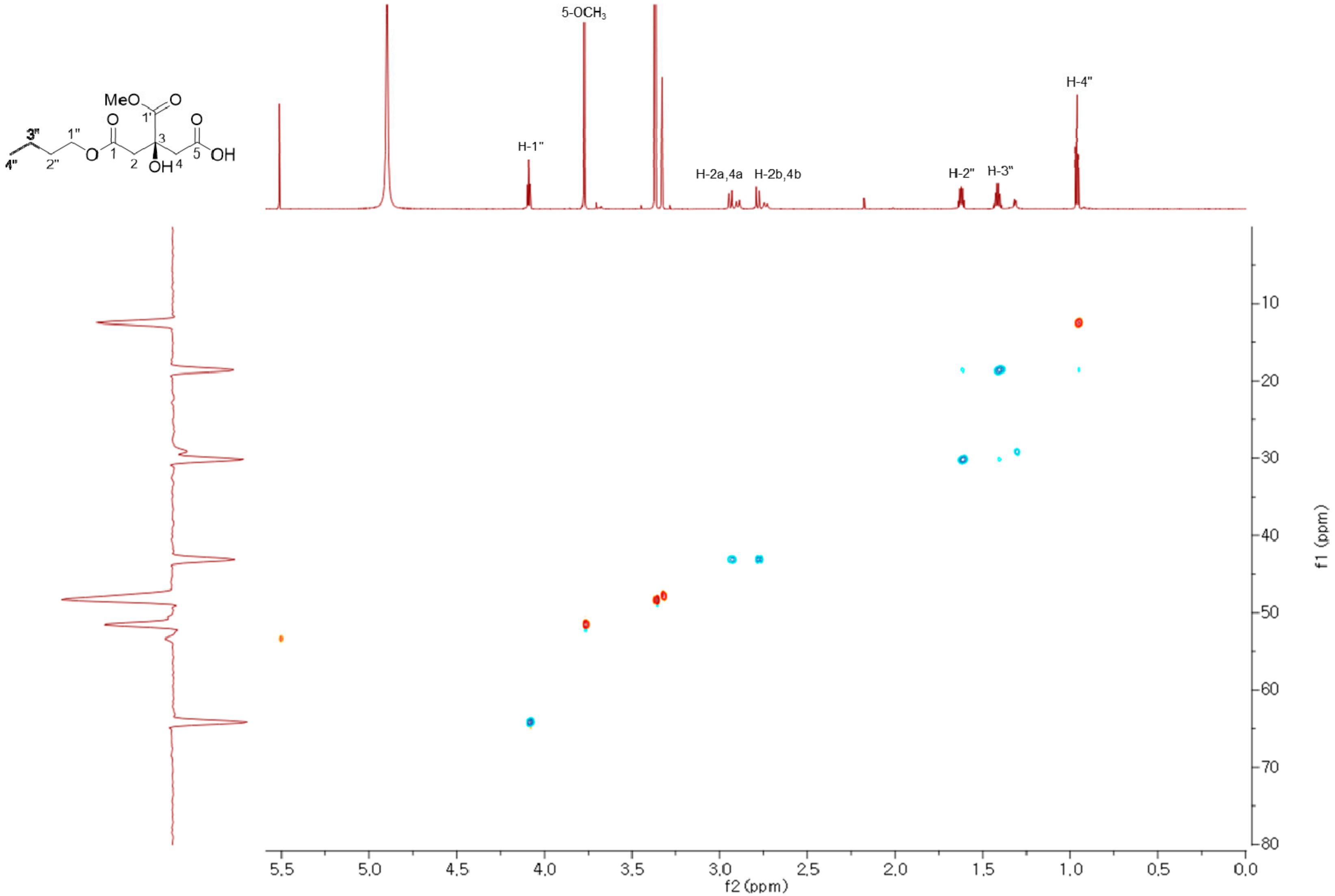

Figure S10. HMBC spectrum of **4** (CD_3OD)

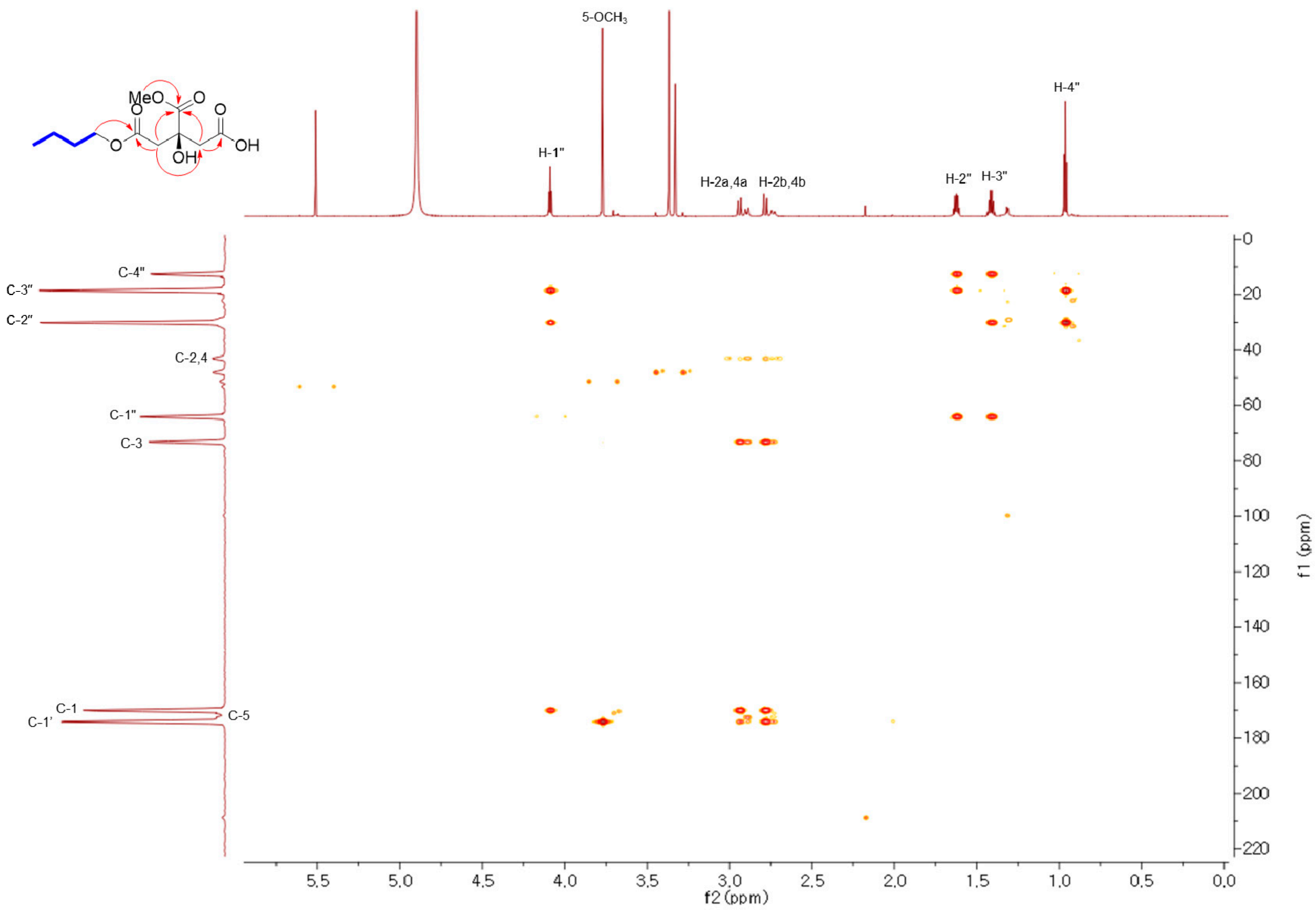

Figure S11. HR-ESIMS data of **6**

Compound Identification Results: Cpd 1277: C11 H18 O7; 4.626

ID Techniques Applied
MFG

Best	Formula	m/z	Mass	Diff (ppm)	RT	Species	Score
⊙	C11 H18 O7	263.1145 280.1401 285.0966	262.1061	3.17	4.626	(M+H)+ (M+NH4)+ (M+Na)+	95.04

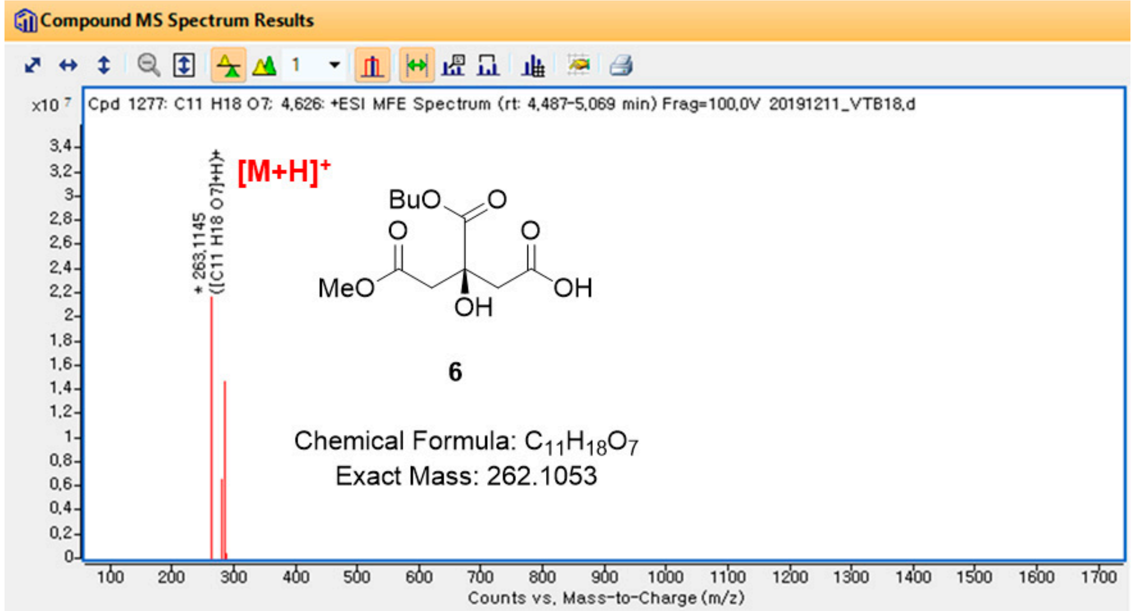

Figure S12. ^{1}H NMR spectrum of **6** (CD_3OD, 700 MHz)

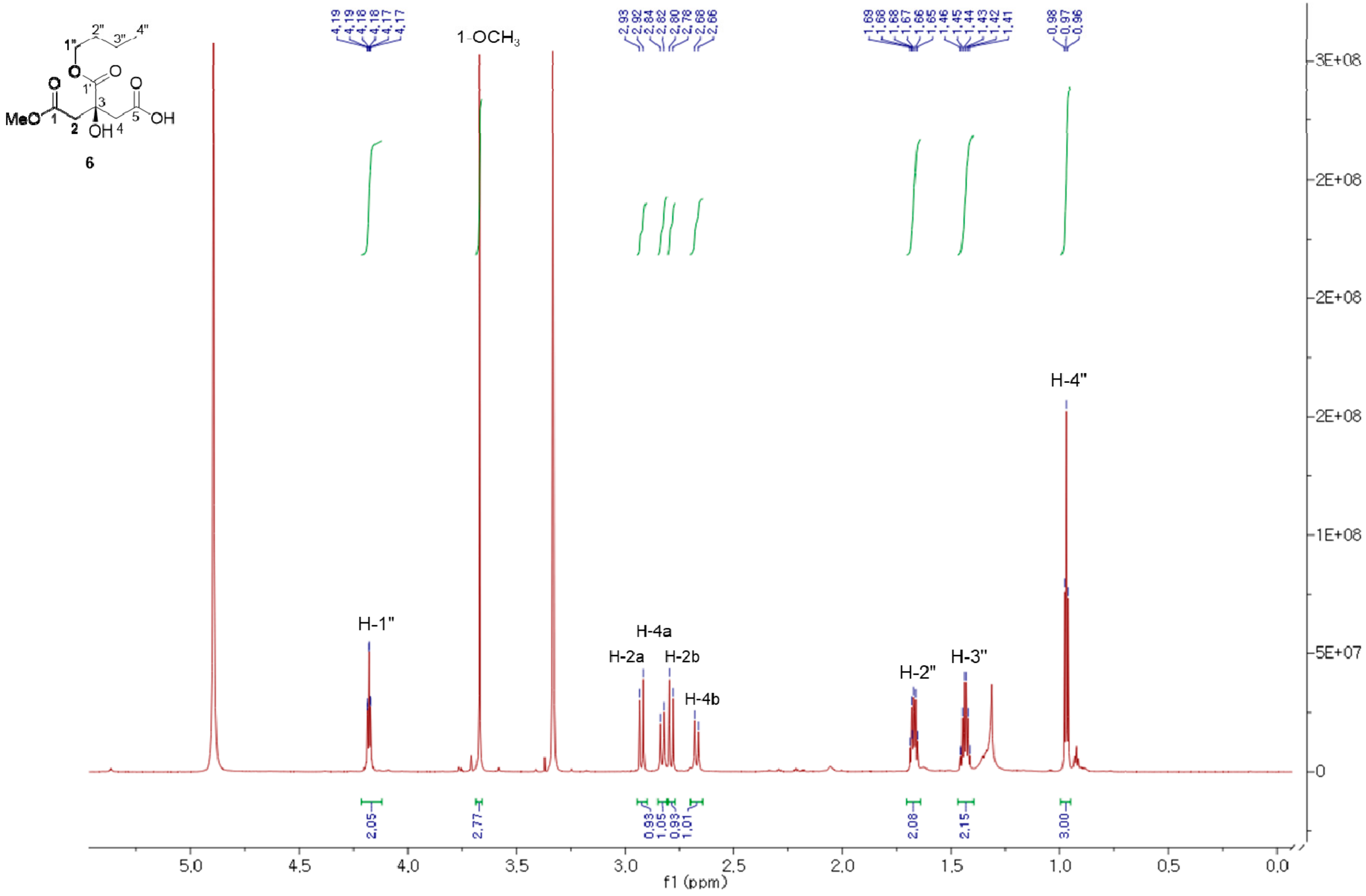

Figure S13. ^{1}H-^{1}H COSY spectrum of **6** (CD_3OD)

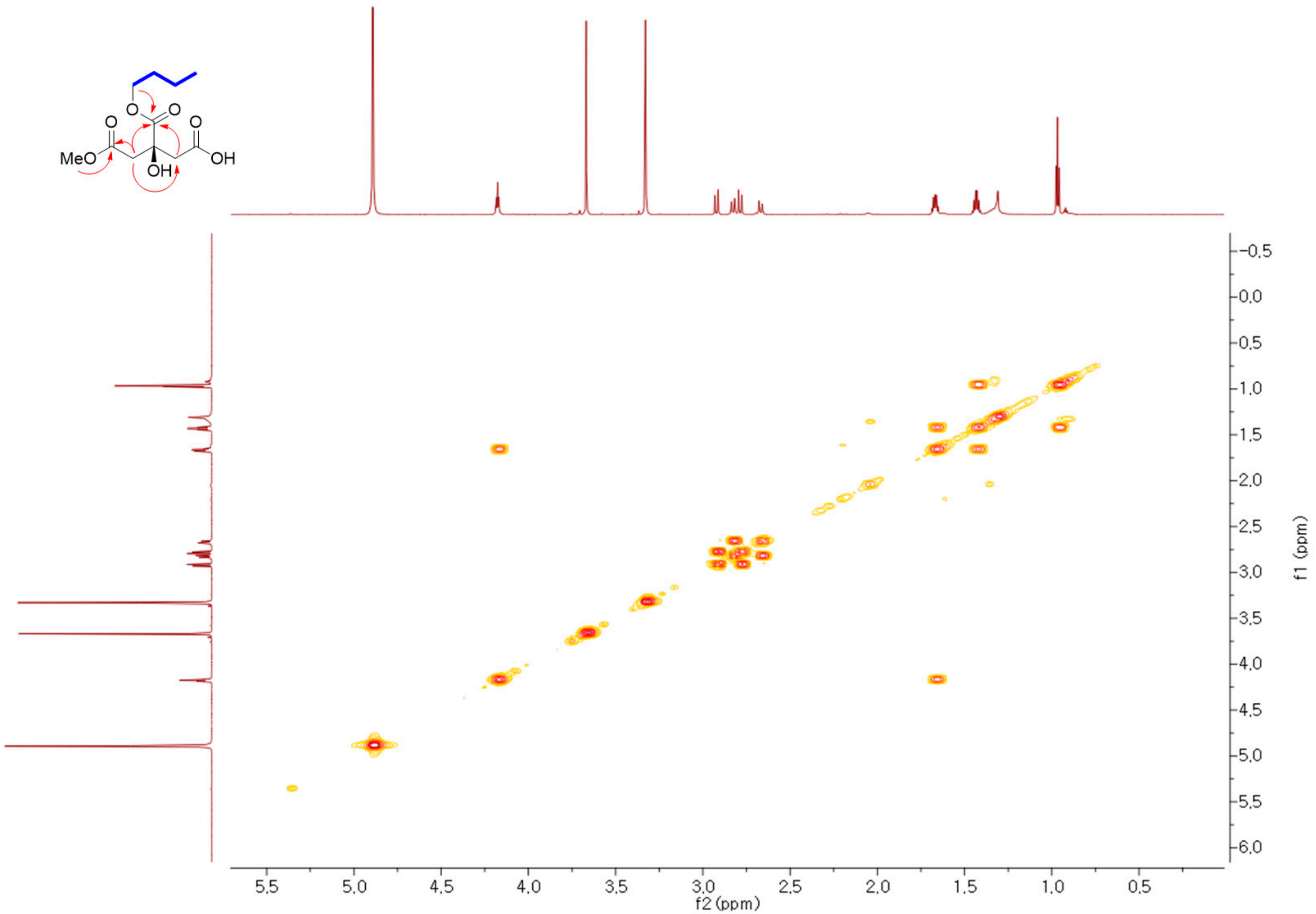

Figure S14. HSQC spectrum of **6** (CD_3OD)

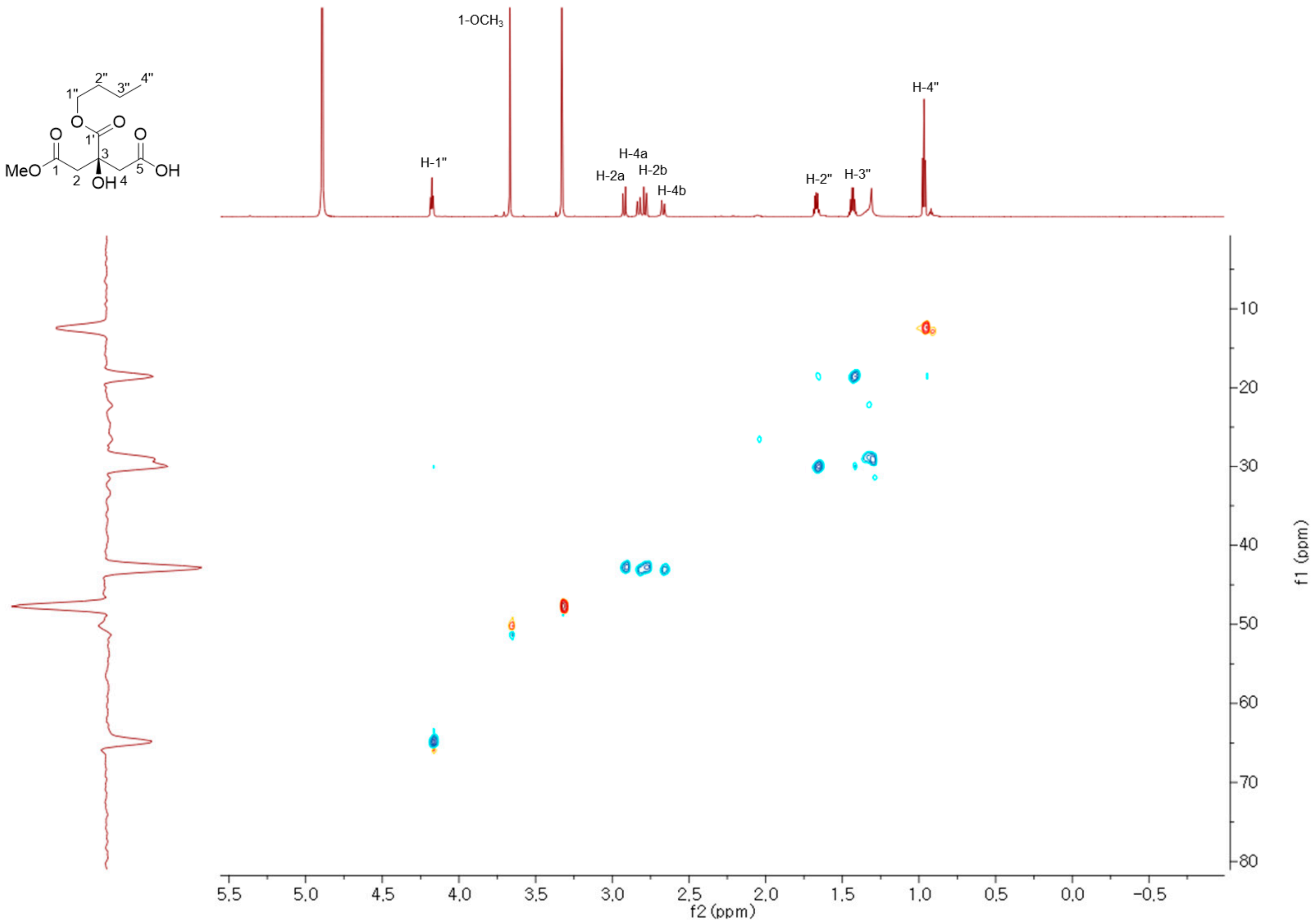

Figure S15. HMBC spectrum of **6** (CD_3OD)

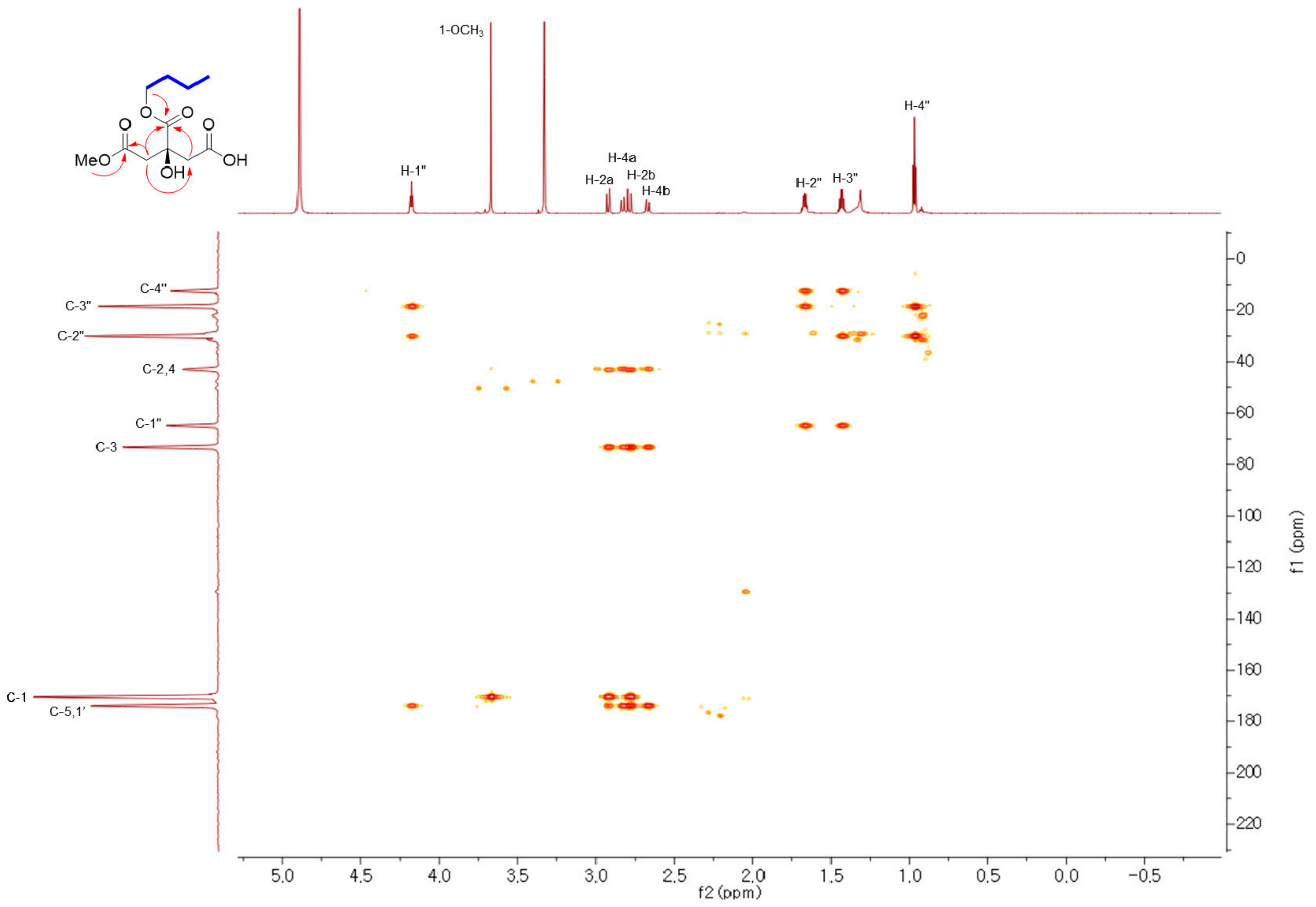

Table S1. Gibbs free energies and Boltzmann distribution of conformers **3*S*** (3*S*)

Conformers	B3LYP/6-31+G(d,p) Gibbs free energy (298.15 K)		
	G (Hartree)	ΔG (kcal/mol)	Boltzmann distribution (%)
**3*S***-1	-956.110424	0.000000	19.98
**3*S***-2	-956.110044	0.000380	13.36
**3*S***-3	-956.109983	0.000442	12.52
**3*S***-4	-956.109973	0.000451	12.39
**3*S***-5	-956.109798	0.000627	10.29
**3*S***-6	-956.109756	0.000669	9.84
**3*S***-7	-956.109704	0.000720	9.32
**3*S***-8	-956.109577	0.000848	8.14
**3*S***-9	-956.108238	0.002186	1.97
**3*S***-10	-956.107726	0.002698	1.15
**3*S***-11	-956.107630	0.002794	1.04

Table S2. Gibbs free energies and Boltzmann distribution of conformers **4*S*** (3*S*)

Conformers	B3LYP/6-31+G(d,p) Gibbs free energy (298.15 K)		
	G (Hartree)	ΔG (kcal/mol)	Boltzmann distribution (%)
**4*S*-**1	-956.110247	0.000000	18.82
**4*S*-**2	-956.109868	0.000379	12.60
**4*S*-**3	-956.109740	0.000507	11.00
**4*S*-**4	-956.109709	0.000538	10.64
**4*S*-**5	-956.109635	0.000612	9.84
**4*S*-**6	-956.109493	0.000754	8.47
**4*S*-**7	-956.109353	0.000893	7.30
**4*S*-**8	-956.109301	0.000946	6.91
**4*S*-**9	-956.109192	0.001055	6.16
**4*S*-**10	-956.109070	0.001176	5.41
**4*S*-**11	-956.108049	0.002198	1.83
**4*S*-**12	-956.107485	0.002762	1.01

Table S3. Gibbs free energies and Boltzmann distribution of conformers ***6S*** (3*S*)

Conformers	B3LYP/6-31+G(d,p) Gibbs free energy (298.15 K)		
	G (Hartree)	ΔG (kcal/mol)	Boltzmann distribution (%)
**6*S*-**1	-956.109734	0.000000	12.75
**6*S*-**2	-956.109664	0.000070	11.84
**6*S*-**3	-956.109658	0.000076	11.77
**6*S*-**4	-956.109638	0.000096	11.52
**6*S*-**5	-956.109595	0.000138	11.02
**6*S*-**6	-956.109560	0.000173	10.62
**6*S*-**7	-956.109418	0.000316	9.12
**6*S*-**8	-956.109281	0.000452	7.90
**6*S*-**9	-956.108367	0.001367	3.00
**6*S*-**10	-956.108325	0.001409	2.87
**6*S*-**11	-956.108098	0.001636	2.25
**6*S*-**12	-956.107791	0.001943	1.63
**6*S*-**13	-956.107610	0.002123	1.35
**6*S*-**14	-956.107603	0.002130	1.34
**6*S*-**15	-956.107359	0.002374	1.03
**6*S*-**16	956.109114	1912.218848	0.00

Coordinates of the conformers

3*S*_1

Center Number	Atomic Coordinates			Atom	Atomic Number	Atomic Type
	X	Y	Z			
1	12.17245817	-5.00920256	8.63639895	c	6	0
2	11.55405016	-2.78397399	6.89025312	c	6	0
3	9.20144385	-3.25827013	5.26006941	c	6	0
4	8.4799967	-1.04471556	3.5742582	c	6	0
5	10.55500591	-0.5987634	1.842868	o	8	0
6	10.32648802	1.42257165	0.31224401	c	6	0
7	12.73500164	1.86698894	-1.1744269	c	6	0
8	14.46026427	3.92081117	0.01587841	c	6	0
9	15.05641171	3.12892111	2.76381002	c	6	0
10	16.99638371	1.49330411	2.92053274	o	8	0
11	13.86553601	3.84098546	4.59103758	o	8	0
12	16.6432132	3.95617343	-1.51131956	o	8	0
13	13.14904681	6.52441201	0.05904854	c	6	0
14	14.87013102	8.54292775	1.12570593	c	6	0
15	13.59192304	10.6041125	1.84088166	o	8	0
16	15.0956883	12.6503633	2.82345741	c	6	0
17	17.16740769	8.36124398	1.28386939	o	8	0
18	8.45308349	2.74070843	0.19832083	o	8	0
19	12.52519746	-6.73907761	7.55200693	h	1	0
20	13.85776961	-4.62910415	9.7759692	h	1	0
21	10.61035586	-5.39850189	9.94041906	h	1	0
22	11.2700899	-1.06296206	8.0147682	h	1	0
23	13.16339774	-2.3988311	5.64374468	h	1	0
24	7.57117064	-3.66305975	6.48032824	h	1	0
25	9.4875817	-4.94329233	4.08367207	h	1	0
26	8.15947329	0.68588985	4.66261357	h	1	0
27	6.77013401	-1.43146394	2.47553063	h	1	0
28	13.8411582	0.13064846	-1.30714273	h	1	0
29	12.24041486	2.50203246	-3.07653706	h	1	0

30	17.163651	1.08204039	4.70409409	h	1	0
31	17.70684527	5.29353426	-0.82722132	h	1	0
32	12.68410323	7.04843819	-1.8911689	h	1	0
33	11.38369533	6.47956022	1.12019085	h	1	0
34	13.75714986	14.14819586	3.27598248	h	1	0
35	16.11282302	12.04585633	4.51554863	h	1	0
36	16.4589544	13.29067446	1.41047696	h	1	0

3*S*_2

Center Number	Atomic Coordinates			Atom	Atomic Number	Atomic Type
	X	Y	Z			
1	9.45803719	-6.65767364	9.11413913	c	6	0
2	8.50618689	-4.36975232	7.61718145	c	6	0
3	10.33981209	-3.55477833	5.52239309	c	6	0
4	9.37017778	-1.28301801	4.05596875	c	6	0
5	11.23241664	-0.66591564	2.14768528	o	8	0
6	10.71167229	1.36328929	0.70314753	c	6	0
7	12.8806535	1.96631163	-1.0699894	c	6	0
8	14.58335848	4.16480522	-0.13442716	c	6	0
9	15.67070667	3.41995848	2.47053404	c	6	0
10	17.85240099	2.12785622	2.29922944	o	8	0
11	14.63905988	3.88544039	4.46618152	o	8	0
12	16.48079113	4.39583302	-1.9903246	o	8	0
13	13.0770918	6.64357341	0.15843494	c	6	0
14	14.770132	8.80542024	0.95504855	c	6	0
15	13.44564785	10.78639028	1.80440425	o	8	0
16	14.9088424	12.95705362	2.55389753	c	6	0
17	17.0753447	8.79257587	0.81561891	o	8	0
18	8.76530196	2.57073511	0.84088065	o	8	0
19	11.27402504	-6.25338548	10.02530056	h	1	0
20	8.11752137	-7.19717868	10.59496671	h	1	0
21	9.72972886	-8.29939188	7.88070718	h	1	0

22	6.65662879	-4.80989961	6.78292558	h	1	0
23	8.18899034	-2.77912285	8.91260566	h	1	0
24	10.6559383	-5.12743213	4.20726953	h	1	0
25	12.18556134	-3.08517325	6.34353711	h	1	0
26	9.1057993	0.36916356	5.27329285	h	1	0
27	7.56313977	-1.67316117	3.12380669	h	1	0
28	14.08675778	0.31413044	-1.33949643	h	1	0
29	12.10197599	2.53476167	-2.89685243	h	1	0
30	18.32470298	1.70875812	4.02542307	h	1	0
31	17.55279087	5.78440924	-1.43643373	h	1	0
32	12.24298695	7.12499355	-1.67619205	h	1	0
33	11.52497758	6.44533187	1.49925703	h	1	0
34	16.17888015	12.46636594	4.10600796	h	1	0
35	16.02595536	13.66083719	0.96600875	h	1	0
36	13.53333174	14.36555265	3.1572081	h	1	0

3*S*_3

Center Number	Atomic Coordinates			Atom	Atomic Number	Atomic Type
	X	Y	Z			
1	12.54228948	-5.19455052	8.46547261	c	6	0
2	11.77215393	-2.91077161	6.86183262	c	6	0
3	9.39882301	-3.40467123	5.26723989	c	6	0
4	8.56762184	-1.16039023	3.67479039	c	6	0
5	10.5542247	-0.63752177	1.8663696	o	8	0
6	10.28639979	1.47999125	0.47684781	c	6	0
7	12.58782849	1.93508848	-1.16691952	c	6	0
8	14.46604568	3.90042607	-0.06038835	c	6	0
9	15.41641042	2.90114527	2.51743222	c	6	0
10	13.88942377	3.62387653	4.45120844	o	8	0
11	17.24818391	1.57712591	2.81453078	o	8	0
12	16.48407076	3.95290481	-1.79376239	o	8	0
13	13.23422275	6.53394031	0.18545725	c	6	0

14	15.08142789	8.46942671	1.19260434	c	6	0
15	13.92285883	10.60995834	1.89525757	o	8	0
16	15.55222431	12.59094929	2.80805742	c	6	0
17	17.36357535	8.16223256	1.32899273	o	8	0
18	8.45257825	2.85461392	0.57684944	o	8	0
19	12.95203928	-6.83775604	7.27302911	h	1	0
20	14.23813308	-4.79436744	9.58164734	h	1	0
21	11.03514595	-5.73484196	9.78025248	h	1	0
22	11.42368923	-1.2782436	8.10023467	h	1	0
23	13.33352645	-2.38319153	5.60638954	h	1	0
24	7.81257588	-3.90995838	6.50744155	h	1	0
25	9.71627986	-5.03410332	4.02352593	h	1	0
26	8.25876402	0.53591135	4.81966655	h	1	0
27	6.81734295	-1.55075542	2.64182037	h	1	0
28	13.63775489	0.18222164	-1.45238499	h	1	0
29	11.95926784	2.652331	-2.99932042	h	1	0
30	14.5733359	2.83270306	5.96264741	h	1	0
31	17.74370974	5.07776899	-1.07000257	h	1	0
32	12.64708515	7.14253729	-1.70654079	h	1	0
33	11.53774555	6.50304486	1.35714859	h	1	0
34	14.30369631	14.16287265	3.26749953	h	1	0
35	16.58185304	11.96346001	4.48446458	h	1	0
36	16.91086007	13.13898238	1.35267522	h	1	0

3*S*_4

Center Number	Atomic Coordinates			Atom	Atomic Number	Atomic Type
	X	Y	Z			
1	5.75474256	-6.35820023	8.18147045	c	6	0
2	8.17902305	-5.78626127	6.70514557	c	6	0
3	8.27255455	-3.06198792	5.71155386	c	6	0
4	10.68245298	-2.53172554	4.24098801	c	6	0
5	10.58291833	0.07255449	3.38799793	o	8	0

6	12.49482346	0.85313839	1.93284968	c	6	0
7	12.16355672	3.56658433	1.08739827	c	6	0
8	14.61584154	4.62280556	-0.08116575	c	6	0
9	16.67856409	4.69341772	1.98327238	c	6	0
10	19.02093776	4.36013453	1.05606483	o	8	0
11	16.24875234	5.08251802	4.20295381	o	8	0
12	15.36379131	3.2076508	-2.21044565	o	8	0
13	14.24981899	7.3532058	-1.08993141	c	6	0
14	13.49544174	9.23582239	0.93271046	c	6	0
15	15.48229938	10.65397934	1.66427365	o	8	0
16	14.98618948	12.37626018	3.70994128	c	6	0
17	11.38933068	9.44115376	1.81642866	o	8	0
18	14.27282986	-0.49614797	1.33517175	o	8	0
19	4.07676596	-6.08940632	6.99745318	h	1	0
20	5.73621594	-8.31054282	8.86607798	h	1	0
21	5.56754407	-5.1117435	9.82459382	h	1	0
22	9.82691174	-6.13538665	7.91841698	h	1	0
23	8.34640449	-7.10739543	5.11247321	h	1	0
24	8.13572475	-1.72627387	7.29163073	h	1	0
25	6.64011211	-2.70035512	4.48466606	h	1	0
26	10.8736913	-3.76519792	2.59065777	h	1	0
27	12.37429379	-2.77854578	5.407396	h	1	0
28	10.67264341	3.61489697	-0.35067124	h	1	0
29	11.51452164	4.71317408	2.67297135	h	1	0
30	20.1547693	4.4976895	2.49624768	h	1	0
31	15.60431827	1.48819822	-1.60237458	h	1	0
32	16.01192875	7.92514838	-1.99820304	h	1	0
33	12.7550097	7.28092325	-2.51482734	h	1	0
34	14.44051853	11.32920742	5.40440554	h	1	0
35	16.75155483	13.39103634	4.02145832	h	1	0
36	13.47068823	13.6852287	3.20627722	h	1	0

3*S*_5

Center Number	Atomic Coordinates			Atom	Atomic Number	Atomic Type
	X	Y	Z			
1	9.29147882	-2.33063416	10.34215878	c	6	0
2	8.64882459	-3.0522986	7.61151626	c	6	0
3	10.37361729	-1.75639042	5.67238453	c	6	0
4	9.72200861	-2.51976028	2.96997775	c	6	0
5	11.43911109	-1.36582889	1.17111697	o	8	0
6	10.79604295	0.9372075	0.29052078	c	6	0
7	12.81550633	1.97835768	-1.45758653	c	6	0
8	14.4821368	4.0622786	-0.24105683	c	6	0
9	15.783546	2.92171572	2.11031183	c	6	0
10	17.95037089	1.71806631	1.55302864	o	8	0
11	14.91835572	3.03130519	4.23330676	o	8	0
12	16.23679909	4.72198642	-2.13525445	o	8	0
13	12.90236244	6.36678428	0.58933658	c	6	0
14	14.5594223	8.42330642	1.68407868	c	6	0
15	13.22619298	10.11400285	3.01120782	o	8	0
16	14.65173172	12.17316439	4.07975878	c	6	0
17	16.84355887	8.57446324	1.37684224	o	8	0
18	8.8585992	2.04014757	0.83510709	o	8	0
19	9.11922001	-0.28775331	10.6339843	h	1	0
20	8.02859541	-3.27028697	11.68540148	h	1	0
21	11.23396974	-2.86717187	10.81968889	h	1	0
22	8.78236646	-5.11370442	7.39260213	h	1	0
23	6.6721552	-2.55737611	7.21385056	h	1	0
24	12.35611592	-2.23266843	6.0490517	h	1	0
25	10.2174927	0.30104548	5.84344669	h	1	0
26	7.78888176	-1.97598268	2.48551547	h	1	0
27	9.95695608	-4.55282436	2.68059619	h	1	0
28	14.06924262	0.47277832	-2.10322755	h	1	0
29	11.88767477	2.82674075	-3.09773204	h	1	0
30	18.56514196	1.02575211	3.14084354	h	1	0
31	17.27301327	6.05805966	-1.40924113	h	1	0
32	11.94608061	7.13595691	-1.08011639	h	1	0

33	11.44169789	5.85014879	1.94679588	h	1	0
34	15.60950889	13.24001216	2.59347537	h	1	0
35	13.27049224	13.35301484	5.04950462	h	1	0
36	16.05989513	11.45786482	5.40969482	h	1	0

3*S*_6

Center Number	Atomic Coordinates			Atom	Atomic Number	Atomic Type
	X	Y	Z			
1	9.55740941	-7.38870908	8.72550547	c	6	0
2	8.76085126	-4.81121905	7.67384142	c	6	0
3	10.45646929	-3.91111137	5.4993021	c	6	0
4	9.63484619	-1.35834945	4.46799493	c	6	0
5	11.35290068	-0.68058041	2.45104067	o	8	0
6	10.84755666	1.50499061	1.24788288	c	6	0
7	12.80569693	2.06379196	-0.76916676	c	6	0
8	14.63424291	4.26685026	-0.12915333	c	6	0
9	16.20623434	3.5169937	2.21538015	c	6	0
10	14.92106622	3.92745885	4.39956966	o	8	0
11	18.30130965	2.62105649	2.15939695	o	8	0
12	16.23559467	4.43966833	-2.24651718	o	8	0
13	13.19875547	6.77067748	0.30028991	c	6	0
14	14.98860422	8.93706534	0.82547396	c	6	0
15	13.75142974	11.01896964	1.57678283	o	8	0
16	15.30143334	13.20453763	2.05795097	c	6	0
17	17.27922516	8.84523672	0.58166939	o	8	0
18	9.04551131	2.84145449	1.73149951	o	8	0
19	11.49634341	-7.33477748	9.45155935	h	1	0
20	8.32061253	-7.9826735	10.27421999	h	1	0
21	9.4794	-8.85120615	7.26080861	h	1	0
22	6.79355047	-4.90932726	7.01887598	h	1	0
23	8.7885963	-3.40302569	9.19970651	h	1	0
24	10.43009738	-5.30074645	3.9594747	h	1	0

25	12.4236841	-3.78111226	6.14323554	h	1	0
26	9.6996096	0.12217178	5.91490709	h	1	0
27	7.7068208	-1.40771877	3.71780676	h	1	0
28	13.95326288	0.39328866	-1.15204355	h	1	0
29	11.81406613	2.59678807	-2.50349639	h	1	0
30	16.01655513	3.33686793	5.75177468	h	1	0
31	17.54541072	5.64513123	-1.79548453	h	1	0
32	12.14860951	7.21655397	-1.42944386	h	1	0
33	11.80739211	6.62433868	1.81528715	h	1	0
34	16.65719052	12.81116777	3.56487482	h	1	0
35	16.33292936	13.75076612	0.35407072	h	1	0
36	13.99535128	14.6923653	2.62479426	h	1	0

3*S*_7

Center Number	Atomic Coordinates			Atom	Atomic Number	Atomic Type
	X	Y	Z			
1	10.39436822	-7.6103645	6.93676111	c	6	0
2	9.73585222	-5.42962647	5.15003171	c	6	0
3	9.59424609	-2.86818208	6.50226632	c	6	0
4	8.93947989	-0.66556024	4.77635508	c	6	0
5	10.98617292	-0.36465768	2.97863452	o	8	0
6	10.66506906	1.44073133	1.21441903	c	6	0
7	12.99621407	1.71810843	-0.43045039	c	6	0
8	14.65591834	4.03644221	0.26202782	c	6	0
9	15.47758538	3.75754586	3.04952588	c	6	0
10	17.65605575	2.47681211	3.31751507	o	8	0
11	14.25694209	4.54639105	4.82480633	o	8	0
12	16.72317229	3.92181146	-1.41490452	o	8	0
13	13.1858426	6.53692975	-0.03173587	c	6	0
14	14.84526243	8.79552439	0.53395867	c	6	0
15	13.49237474	10.90615456	0.87398596	o	8	0
16	14.92602949	13.16750807	1.36867318	c	6	0

17	17.15169602	8.74137391	0.63775655	o	8	0
18	8.75936409	2.69994324	0.99971327	o	8	0
19	8.9845941	-7.80217319	8.44307173	h	1	0
20	10.48045941	-9.41319763	5.92469076	h	1	0
21	12.23447792	-7.30895684	7.83970981	h	1	0
22	11.14302931	-5.31578501	3.6335374	h	1	0
23	7.91548538	-5.81107293	4.22622421	h	1	0
24	11.3954908	-2.46560733	7.44967817	h	1	0
25	8.15097972	-2.93975711	7.99343003	h	1	0
26	8.7368439	1.10552467	5.82524658	h	1	0
27	7.18253849	-0.99164237	3.73204129	h	1	0
28	14.18593239	0.03743429	-0.3021885	h	1	0
29	12.39483415	1.96430694	-2.39185675	h	1	0
30	17.95447097	2.36718862	5.12796933	h	1	0
31	17.77308051	5.37688684	-1.01061578	h	1	0
32	12.53981539	6.69061833	-1.99435907	h	1	0
33	11.51006384	6.58611888	1.16599094	h	1	0
34	13.53164111	14.66960032	1.56790229	h	1	0
35	16.02334036	12.957687	3.10498022	h	1	0
36	16.2117525	13.5670114	-0.19711735	h	1	0

3*S*_8

Center Number	Atomic Coordinates			Atom	Atomic Number	Atomic Type
	X	Y	Z			
1	12.28973763	-1.97350498	9.14831118	c	6	0
2	11.51179957	-2.84235872	6.49888461	c	6	0
3	9.1848358	-1.43201112	5.49413678	c	6	0
4	8.55715086	-2.0352557	2.7457347	c	6	0
5	10.5930374	-1.20681225	1.09951801	o	8	0
6	10.36840902	1.10760234	0.05433984	c	6	0
7	12.74161446	1.81732843	-1.38836306	c	6	0
8	14.40955093	3.80295319	-0.01595376	c	6	0

9	15.1314323	2.73435667	2.60461727	c	6	0
10	17.1529684	1.19919376	2.52355451	o	8	0
11	13.9615484	3.16792778	4.53275857	o	8	0
12	16.53960837	4.10469162	-1.58867844	o	8	0
13	13.00129924	6.32623825	0.36153248	c	6	0
14	14.68007532	8.27910558	1.60382974	c	6	0
15	13.35256054	10.20956349	2.55534231	o	8	0
16	14.81143906	12.19066318	3.72224325	c	6	0
17	16.98466692	8.15803836	1.70435519	o	8	0
18	8.52261648	2.45570473	0.25767884	o	8	0
19	10.74000836	-2.20254282	10.50483548	h	1	0
20	13.90288737	-3.06120141	9.8569814	h	1	0
21	12.82561797	0.02541196	9.12632626	h	1	0
22	13.08873928	-2.55408337	5.18617956	h	1	0
23	11.12634124	-4.88345488	6.50534894	h	1	0
24	9.46851536	0.61304413	5.66701675	h	1	0
25	7.52183772	-1.90403138	6.64499589	h	1	0
26	6.80978066	-1.11489255	2.14782575	h	1	0
27	8.41727384	-4.07259062	2.42260295	h	1	0
28	13.91032486	0.15361469	-1.73412333	h	1	0
29	12.18990288	2.63853818	-3.20270702	h	1	0
30	17.40145394	0.59594974	4.24202591	h	1	0
31	17.57821158	5.38965418	-0.77802433	h	1	0
32	12.44913976	7.04836882	-1.50030043	h	1	0
33	11.27441756	6.08623308	1.45964598	h	1	0
34	13.43637836	13.58861617	4.35053701	h	1	0
35	15.87949859	11.44693853	5.32507326	h	1	0
36	16.12624449	13.01924628	2.36214184	h	1	0

3*S*_9

Center Number	Atomic Coordinates			Atom	Atomic Number	Atomic Type
	X	Y	Z			
1	6.24570534	-3.51646528	9.23397318	c	6	0
2	8.52154206	-2.74075059	7.62187976	c	6	0
3	8.45123839	-3.8859315	4.95860708	c	6	0
4	10.68816931	-3.16082789	3.30798781	c	6	0
5	10.58210385	-0.4492538	2.88883298	o	8	0
6	12.37854073	0.53417612	1.41025473	c	6	0
7	12.04486215	3.35178141	1.04074498	c	6	0
8	14.42994449	4.5628538	-0.11794991	c	6	0
9	16.63631668	4.26950545	1.77073883	c	6	0
10	18.89511887	4.03330623	0.63161767	o	8	0
11	16.37694981	4.31802607	4.04998265	o	8	0
12	14.99127724	3.51335325	-2.50119125	o	8	0
13	14.05124069	7.4264003	-0.63289049	c	6	0
14	13.48741708	8.95815767	1.72063755	c	6	0
15	15.54298229	10.24594358	2.5011038	o	8	0
16	15.23376908	11.62617043	4.8235042	c	6	0
17	11.46247822	9.01746273	2.79570647	o	8	0
18	14.06159601	-0.7222709	0.44655792	o	8	0
19	6.14921504	-5.57373631	9.4618365	h	1	0
20	6.34325096	-2.67911616	11.12324074	h	1	0
21	4.46845473	-2.9003841	8.36604249	h	1	0
22	8.60360366	-0.67702313	7.46871206	h	1	0
23	10.27664283	-3.32473427	8.56615655	h	1	0
24	6.69883684	-3.3332466	3.99467357	h	1	0
25	8.42341094	-5.95790794	5.08488061	h	1	0
26	10.64006727	-4.11132962	1.47212632	h	1	0
27	12.49692354	-3.61208516	4.2073912	h	1	0
28	10.45985875	3.64581551	-0.26043189	h	1	0
29	11.53243803	4.23263022	2.83239164	h	1	0
30	20.13168377	3.92159306	1.98710635	h	1	0

31	15.22861072	1.71285945	-2.20689919	h	1	0
32	15.75349425	8.12407508	-1.56662926	h	1	0
33	12.45478197	7.60499918	-1.9324621	h	1	0
34	17.02441917	12.5952056	5.13631713	h	1	0
35	13.68718273	12.98484405	4.66032787	h	1	0
36	14.83028678	10.3250489	6.37555119	h	1	0

3*S*_10

Center Number	Atomic Coordinates			Atom	Atomic Number	Atomic Type
	X	Y	Z			
1	4.65000254	-4.37980623	6.91775054	c	6	0
2	6.79302649	-3.66879881	5.10601411	c	6	0
3	9.39794386	-3.69007035	6.38176185	c	6	0
4	11.56758267	-3.01133783	4.62455767	c	6	0
5	11.21632753	-0.41021759	3.82004385	o	8	0
6	12.87746969	0.48947279	2.14345276	c	6	0
7	12.29015296	3.1869757	1.39191183	c	6	0
8	14.54810315	4.42601314	0.02720744	c	6	0
9	16.7926146	4.5777219	1.88763806	c	6	0
10	19.05068899	4.44347515	0.72974603	o	8	0
11	16.55585941	4.86135617	4.15186952	o	8	0
12	15.17239027	3.11246356	-2.20415898	o	8	0
13	13.91877408	7.15127429	-0.85761034	c	6	0
14	13.24550306	8.92929848	1.28493414	c	6	0
15	15.20055747	10.45555652	1.87023997	o	8	0
16	14.79581361	12.09322046	4.00341098	c	6	0
17	11.22516758	8.97395242	2.36938952	o	8	0
18	14.63565962	-0.75124306	1.30173591	o	8	0
19	4.54729224	-3.05929041	8.51036496	h	1	0
20	2.81604582	-4.35116947	5.9601567	h	1	0
21	4.92237712	-6.27984264	7.69818261	h	1	0
22	6.81258743	-4.98437694	3.4990579	h	1	0

23	6.44554546	-1.78676068	4.31296696	h	1	0
24	9.79093646	-5.57953778	7.14737041	h	1	0
25	9.4074662	-2.38806966	7.99651953	h	1	0
26	11.61692319	-4.21973261	2.94526283	h	1	0
27	13.40327945	-3.15842589	5.56576665	h	1	0
28	10.67553839	3.15529883	0.09376101	h	1	0
29	11.71422585	4.26140046	3.05384319	h	1	0
30	20.30800881	4.61871137	2.05911983	h	1	0
31	15.55537781	1.39205161	-1.67582068	h	1	0
32	15.54696051	7.859014	-1.90886578	h	1	0
33	12.30155491	7.01822767	-2.13654817	h	1	0
34	13.15333283	13.30421129	3.68812432	h	1	0
35	14.49285383	10.9735223	5.71217107	h	1	0
36	16.51125371	13.2213235	4.17029026	h	1	0

3*S*_11

Center Number	Atomic Coordinates			Atom	Atomic Number	Atomic Type
	X	Y	Z			
1	6.37764163	-6.52974416	8.62272429	c	6	0
2	8.51859718	-5.95420559	6.75966091	c	6	0
3	8.65041176	-3.14877268	6.0331356	c	6	0
4	10.77906582	-2.60958197	4.18029334	c	6	0
5	10.73270457	0.07240216	3.61698393	o	8	0
6	12.43318973	0.89706872	1.9320698	c	6	0
7	12.15542699	3.69991952	1.42957371	c	6	0
8	14.33041639	4.72092178	-0.22499205	c	6	0
9	16.9226748	4.37406771	1.07470298	c	6	0
10	16.82623747	4.96057037	3.57169831	o	8	0
11	18.83919326	3.72206095	0.02730662	o	8	0
12	14.36501752	3.51695224	-2.59722592	o	8	0
13	13.97570401	7.56515305	-0.85275345	c	6	0
14	13.85165152	9.29462708	1.42717245	c	6	0

15	15.9569779	10.71822287	1.62839766	o	8	0
16	16.02989502	12.37782184	3.77903144	c	6	0
17	12.09584342	9.41371157	2.89810218	o	8	0
18	13.98937374	-0.47136064	0.91924167	o	8	0
19	4.53481341	-6.02507312	7.82362081	h	1	0
20	6.32519459	-8.54040165	9.10713229	h	1	0
21	6.60865357	-5.46241474	10.38247249	h	1	0
22	10.3308506	-6.53967927	7.58568468	h	1	0
23	8.27129608	-7.09949545	5.04599897	h	1	0
24	8.92071035	-1.98924761	7.73119265	h	1	0
25	6.85315043	-2.55162967	5.1881676	h	1	0
26	10.55850966	-3.66445443	2.41351975	h	1	0
27	12.63268939	-3.0906509	4.96551512	h	1	0
28	10.37571036	3.99064834	0.40894131	h	1	0
29	11.98662963	4.71916799	3.2144634	h	1	0
30	18.53987429	4.76249318	4.20582796	h	1	0
31	14.83184453	1.77100729	-2.27078634	h	1	0
32	15.51501444	8.12422743	-2.10652786	h	1	0
33	12.1916646	7.72945093	-1.88474358	h	1	0
34	14.42784736	13.68095293	3.74731825	h	1	0
35	15.96652837	11.2858928	5.53157473	h	1	0
36	17.80751475	13.40739277	3.62713485	h	1	0

4*S*_1

Center Number	Atomic Coordinates			Atom	Atomic Number	Atomic Type
	X	Y	Z			
1	-9.19247153	-7.37828436	-2.43993307	c	6	0
2	-6.5711661	-6.81035526	-1.35104296	c	6	0
3	-6.49354305	-6.98746226	1.54266579	c	6	0
4	-3.92802692	-6.38565218	2.68838655	c	6	0
5	-2.14703806	-8.28079245	1.82666507	o	8	0
6	0.2413181	-8.01811413	2.66312537	c	6	0

7	1.97805155	-9.99042795	1.51957459	c	6	0
8	3.75374579	-8.94549319	-0.5661925	c	6	0
9	2.09496995	-7.85181724	-2.71025938	c	6	0
10	1.70322685	-9.51324698	-4.58025291	o	8	0
11	0.14253426	-8.61660668	-6.61871775	c	6	0
12	1.20404982	-5.73621567	-2.65245636	o	8	0
13	5.18861643	-11.0507233	-1.34891664	o	8	0
14	5.47083731	-6.81812937	0.44785039	c	6	0
15	7.30153398	-5.91657601	-1.54521322	c	6	0
16	8.56531146	-3.83042467	-0.83518161	o	8	0
17	7.66237427	-6.93602898	-3.58191863	o	8	0
18	0.90981916	-6.39630016	4.14248521	o	8	0
19	-9.81541954	-9.28941816	-1.93858918	h	1	0
20	-10.61130841	-6.05139796	-1.71849353	h	1	0
21	-9.1985479	-7.23834479	-4.50482713	h	1	0
22	-5.1795131	-8.12709153	-2.14053619	h	1	0
23	-5.96545979	-4.91244533	-1.93280549	h	1	0
24	-7.07851864	-8.88266636	2.1519839	h	1	0
25	-7.86353117	-5.65934279	2.36193461	h	1	0
26	-3.97782749	-6.41811333	4.7560925	h	1	0
27	-3.23298938	-4.52750873	2.09957841	h	1	0
28	3.18202577	-10.75016292	3.01834939	h	1	0
29	0.8876503	-11.53826056	0.69895346	h	1	0
30	0.03284116	-10.17746556	-7.95811796	h	1	0
31	0.9955126	-6.95608228	-7.50158371	h	1	0
32	-1.73756241	-8.11585769	-5.92541166	h	1	0
33	6.24005836	-10.45728177	-2.73338241	h	1	0
34	6.56999312	-7.54300672	2.04738936	h	1	0
35	4.37361521	-5.21886397	1.14820585	h	1	0
36	9.70905383	-3.41434599	-2.21302063	h	1	0

4*S*_2

Center Number	Atomic Coordinates			Atom	Atomic Number	Atomic Type
	X	Y	Z			
1	-11.21864995	-4.95497586	2.77669754	c	6	0
2	-9.36171061	-6.88272403	1.67234693	c	6	0
3	-6.5936575	-6.05850554	1.94313524	c	6	0
4	-4.7780404	-7.99600598	0.84676175	c	6	0
5	-2.23191054	-7.03716798	1.18517621	o	8	0
6	-0.35373456	-8.53405127	0.40208718	c	6	0
7	2.21910555	-7.38359067	0.89762342	c	6	0
8	4.29890874	-8.74653478	-0.62370956	c	6	0
9	3.77488908	-8.36037402	-3.4753452	c	6	0
10	4.67435015	-10.26197532	-4.88433957	o	8	0
11	4.34466481	-9.96887383	-7.56758958	c	6	0
12	2.73490023	-6.49973616	-4.32830349	o	8	0
13	4.45391619	-11.32121349	0.04430618	o	8	0
14	6.96323149	-7.66965556	-0.03001831	c	6	0
15	7.25812226	-4.89366167	-0.65501374	c	6	0
16	8.51028964	-4.53585347	-2.85536031	o	8	0
17	6.47317254	-3.15855452	0.61866651	o	8	0
18	-0.6974858	-10.60558094	-0.56282967	o	8	0
19	-10.87534574	-4.65769681	4.79752397	h	1	0
20	-11.0405499	-3.11903288	1.83479891	h	1	0
21	-13.17598538	-5.58799855	2.5569896	h	1	0
22	-9.63077017	-8.7164307	2.60835277	h	1	0
23	-9.7963359	-7.19099521	-0.33393121	h	1	0
24	-6.13655734	-5.76028429	3.94390376	h	1	0
25	-6.2991518	-4.24097778	0.98896115	h	1	0
26	-5.10153157	-8.30670453	-1.17368228	h	1	0
27	-4.93044848	-9.82933297	1.79509377	h	1	0
28	2.16471506	-5.36437184	0.48937393	h	1	0
29	2.63694131	-7.59313131	2.91642432	h	1	0
30	2.33909903	-9.81906427	-8.03629609	h	1	0
31	5.16497957	-11.65838174	-8.41289356	h	1	0
32	5.31810398	-8.27257153	-8.23237524	h	1	0

33	2.78703787	-12.01949181	-0.3015777	h	1	0
34	8.33653759	-8.80106307	-1.07388943	h	1	0
35	7.28727676	-7.92056241	1.99473462	h	1	0
36	8.52778319	-2.72066763	-3.14616428	h	1	0

4*S*_3

Center Number	Atomic Coordinates			Atom	Atomic Number	Atomic Type
	X	Y	Z			
1	-9.54946294	-8.60984804	-2.1804906	c	6	0
2	-6.96178181	-7.5996092	-1.36510884	c	6	0
3	-6.72588797	-7.29299704	1.50917951	c	6	0
4	-4.1747559	-6.29530064	2.38035195	c	6	0
5	-2.27261804	-8.15006477	1.71879617	o	8	0
6	0.11770869	-7.58890616	2.39715335	c	6	0
7	1.94586433	-9.63672895	1.57200551	c	6	0
8	3.87227321	-8.84844851	-0.51910199	c	6	0
9	2.40329228	-8.20236181	-2.94688986	c	6	0
10	1.24788372	-5.95859454	-2.84489528	o	8	0
11	-0.11405416	-5.24126014	-5.09178526	c	6	0
12	2.22933565	-9.69123055	-4.68598493	o	8	0
13	5.40290031	-10.97045304	-1.04623352	o	8	0
14	5.58992882	-6.69052588	0.37879211	c	6	0
15	7.34635195	-5.78760679	-1.68376351	c	6	0
16	9.50521702	-4.81027865	-0.73447054	o	8	0
17	6.90508188	-5.84449961	-3.93388748	o	8	0
18	0.70259694	-5.68322746	3.5353337	o	8	0
19	-9.92207404	-10.47015973	-1.34858715	h	1	0
20	-11.07560597	-7.34220898	-1.58336248	h	1	0
21	-9.66619932	-8.80912162	-4.23667904	h	1	0
22	-5.47036865	-8.87457182	-2.03066978	h	1	0
23	-6.61348669	-5.76378366	-2.27352216	h	1	0
24	-7.0908941	-9.10907475	2.44415016	h	1	0

25	-8.17703113	-5.97629679	2.19535819	h	1	0
26	-4.12055425	-5.99067908	4.42530291	h	1	0
27	-3.68791096	-4.5010041	1.46767959	h	1	0
28	3.05961066	-10.20656586	3.21888653	h	1	0
29	0.90934472	-11.28486853	0.88756004	h	1	0
30	-1.6159998	-6.60042747	-5.48997554	h	1	0
31	1.18163066	-5.15652894	-6.69627145	h	1	0
32	-0.89943681	-3.38045577	-4.69031482	h	1	0
33	4.81993766	-11.61000475	-2.67048493	h	1	0
34	6.70701807	-7.35324663	1.98588107	h	1	0
35	4.46540503	-5.08757681	1.04617678	h	1	0
36	10.48177002	-4.22496681	-2.17751786	h	1	0

4*S*_4

Center Number	Atomic Coordinates			Atom	Atomic Number	Atomic Type
	X	Y	Z			
1	-11.45371947	-7.35375624	-0.47803462	c	6	0
2	-9.00835946	-6.13954895	0.48619681	c	6	0
3	-6.71079673	-7.8941925	0.24730703	c	6	0
4	-4.30381339	-6.66852661	1.22755332	c	6	0
5	-2.26135527	-8.45853292	0.90544567	o	8	0
6	0.00281515	-7.73515053	1.81947237	c	6	0
7	1.9978769	-9.74653121	1.38125215	c	6	0
8	4.12390379	-9.0048076	-0.49635596	c	6	0
9	2.9575522	-8.65806322	-3.15376994	c	6	0
10	1.78572075	-6.39911643	-3.36961171	o	8	0
11	0.57726021	-5.94566259	-5.76469809	c	6	0
12	3.03233367	-10.22291673	-4.81352868	o	8	0
13	5.75047551	-11.11364592	-0.5239243	o	8	0
14	5.54865415	-6.61469344	0.37472167	c	6	0
15	7.69789123	-5.96322587	-1.38281374	c	6	0
16	8.88870642	-3.81901417	-0.69368422	o	8	0

17	8.34249768	-7.1907115	-3.218793	o	8	0
18	0.36639083	-5.73358599	2.88179696	o	8	0
19	-11.29561688	-7.87114012	-2.47727019	h	1	0
20	-11.90074359	-9.07784813	0.57943197	h	1	0
21	-13.05941241	-6.06343989	-0.28575351	h	1	0
22	-8.64775011	-4.38314054	-0.56099298	h	1	0
23	-9.2480468	-5.5849318	2.47230926	h	1	0
24	-6.43793557	-8.43662286	-1.73603484	h	1	0
25	-7.04936559	-9.65003513	1.29840986	h	1	0
26	-4.44772268	-6.16980002	3.23088491	h	1	0
27	-3.83604826	-4.93706945	0.19148128	h	1	0
28	2.9141832	-10.14317468	3.19337609	h	1	0
29	1.13324422	-11.48886824	0.69570489	h	1	0
30	-0.90362792	-7.34555152	-6.10039098	h	1	0
31	1.95789088	-6.04969418	-7.29640796	h	1	0
32	-0.22453785	-4.05283835	-5.63323253	h	1	0
33	6.82900215	-10.9156027	-1.99366276	h	1	0
34	6.3454111	-6.96773382	2.25393433	h	1	0
35	4.29359342	-4.9879179	0.56908719	h	1	0
36	10.24780888	-3.5671779	-1.90544486	h	1	0

4*S*_5

Center Number	Atomic Coordinates			Atom	Atomic Number	Atomic Type
	X	Y	Z			
1	-10.80314044	-6.29949635	-1.04474633	c	6	0
2	-8.4940577	-5.64320249	0.57336875	c	6	0
3	-6.27607137	-7.46570157	0.15849386	c	6	0
4	-3.99458771	-6.78124407	1.76384428	c	6	0
5	-2.0266186	-8.60759464	1.23597417	o	8	0
6	0.20976418	-8.19101284	2.37665714	c	6	0
7	2.19129146	-10.06931783	1.50690222	c	6	0
8	4.00420749	-8.99733477	-0.53427785	c	6	0

9	2.4016405	-8.1776524	-2.83946538	c	6	0
10	2.09771838	-10.04862235	-4.51766562	o	8	0
11	0.55656262	-9.43270385	-6.67066004	c	6	0
12	1.47469956	-6.0877696	-3.05225074	o	8	0
13	5.65583282	-11.0108981	-1.10080027	o	8	0
14	5.46741992	-6.67435678	0.44646262	c	6	0
15	7.32990121	-5.72009403	-1.49281387	c	6	0
16	8.27376567	-3.43814136	-0.89900173	o	8	0
17	7.97648679	-6.85994648	-3.39049554	o	8	0
18	0.58756459	-6.49797076	3.87866079	o	8	0
19	-10.34466171	-6.23808346	-3.06388667	h	1	0
20	-11.49888018	-8.20362766	-0.61955086	h	1	0
21	-12.35592811	-4.97318062	-0.71203307	h	1	0
22	-7.88153646	-3.70532951	0.15078532	h	1	0
23	-9.02942495	-5.65347089	2.57916514	h	1	0
24	-5.71626525	-7.45295434	-1.83867043	h	1	0
25	-6.86198813	-9.40571724	0.60135484	h	1	0
26	-4.4260575	-6.83205578	3.78836466	h	1	0
27	-3.27478645	-4.89291347	1.32106859	h	1	0
28	3.34765132	-10.60905705	3.13183163	h	1	0
29	1.30355621	-11.75979316	0.72515999	h	1	0
30	1.37367929	-7.84888705	-7.71407637	h	1	0
31	-1.35340156	-8.92567735	-6.06913223	h	1	0
32	0.52628206	-11.1324267	-7.83332667	h	1	0
33	6.77598018	-10.38633392	-2.4171374	h	1	0
34	6.53187639	-7.21886184	2.13892915	h	1	0
35	4.19990464	-5.1438566	0.99491629	h	1	0
36	9.46920459	-3.0017875	-2.22596741	h	1	0

4*S*_6

Center Number	Atomic Coordinates			Atom	Atomic Number	Atomic Type
	X	Y	Z			

1	-9.19087224	-2.99322054	1.51212366	c	6	0
2	-7.14143166	-4.82127632	0.59617857	c	6	0
3	-6.80607647	-7.1027436	2.35448826	c	6	0
4	-4.78902395	-8.95970633	1.49483112	c	6	0
5	-2.36795322	-7.66776837	1.57399092	o	8	0
6	-0.34795978	-9.01151506	0.87309401	c	6	0
7	2.07598204	-7.50635149	1.09160234	c	6	0
8	4.22764737	-8.73441094	-0.44386742	c	6	0
9	3.4890032	-8.71021724	-3.27413233	c	6	0
10	4.61309682	-10.56940375	-4.57420034	o	8	0
11	4.09846752	-10.58785182	-7.24452482	c	6	0
12	2.1089268	-7.13134794	-4.20843028	o	8	0
13	4.76928326	-11.18850707	0.44311989	o	8	0
14	6.75064213	-7.26627974	-0.14409212	c	6	0
15	6.62403906	-4.55654785	-1.05135774	c	6	0
16	7.68716432	-4.26909528	-3.35919655	o	8	0
17	5.6774834	-2.81651503	0.09973687	o	8	0
18	-0.46215246	-11.2143935	0.18359662	o	8	0
19	-11.03420933	-3.93064613	1.64320783	h	1	0
20	-8.73850861	-2.24020142	3.38836931	h	1	0
21	-9.39357012	-1.38638354	0.22477883	h	1	0
22	-7.61027389	-5.49669143	-1.31056036	h	1	0
23	-5.33341724	-3.82447163	0.43052785	h	1	0
24	-8.59245695	-8.15166841	2.49451973	h	1	0
25	-6.35174209	-6.45824062	4.27356363	h	1	0
26	-5.11369016	-9.62040874	-0.43947951	h	1	0
27	-4.68745737	-10.62101609	2.72267081	h	1	0
28	1.73668819	-5.55621784	0.5204512	h	1	0
29	2.63274511	-7.4781153	3.08751457	h	1	0
30	2.07203063	-10.78743314	-7.58856336	h	1	0
31	5.12681759	-12.20719022	-7.99439818	h	1	0
32	4.75859428	-8.83564198	-8.11689221	h	1	0
33	3.18700521	-12.11918699	0.31047422	h	1	0
34	8.20261784	-8.3117847	-1.17083248	h	1	0
35	7.22123094	-7.26047675	1.86735994	h	1	0

36	7.43576108	-2.50818655	-3.82375438	h	1	0

4*S*_7

Center Number	Atomic Coordinates			Atom	Atomic Number	Atomic Type
	X	Y	Z			
1	-9.51691322	-8.82586414	-2.19101245	c	6	0
2	-6.94568862	-7.76128493	-1.39341963	c	6	0
3	-6.71639156	-7.39883159	1.47490578	c	6	0
4	-4.18278862	-6.34285813	2.32813811	c	6	0
5	-2.24902183	-8.17597978	1.69881265	o	8	0
6	0.13073229	-7.56388659	2.37028624	c	6	0
7	1.99404295	-9.59361983	1.57907903	c	6	0
8	3.91782542	-8.80225144	-0.51296856	c	6	0
9	2.4513767	-8.21205866	-2.95668738	c	6	0
10	1.25065198	-5.99109144	-2.88624806	o	8	0
11	-0.11260871	-5.32634087	-5.14848916	c	6	0
12	2.31773618	-9.72302272	-4.68031156	o	8	0
13	5.48523723	-10.90600385	-1.00443738	o	8	0
14	5.59620261	-6.6049785	0.36464974	c	6	0
15	7.35091379	-5.70444287	-1.70035757	c	6	0
16	9.50085879	-4.70496196	-0.75379825	o	8	0
17	6.91459457	-5.78044391	-3.95090326	o	8	0
18	0.68201093	-5.63121472	3.47937629	o	8	0
19	-9.6289831	-9.0654535	-4.24316258	h	1	0
20	-9.86168684	-10.67571718	-1.32446181	h	1	0
21	-11.06268959	-7.57069548	-1.61862197	h	1	0
22	-5.43445012	-9.02504376	-2.03505712	h	1	0
23	-6.62517447	-5.93729015	-2.33506754	h	1	0
24	-7.05148958	-9.20354359	2.44242085	h	1	0
25	-8.18954668	-6.09423157	2.13689766	h	1	0
26	-4.1340381	-6.00129441	4.36736674	h	1	0
27	-3.7268933	-4.55680438	1.38365105	h	1	0

28	3.10889434	-10.12534085	3.23799893	h	1	0
29	0.98603491	-11.26624855	0.91176671	h	1	0
30	-1.57713518	-6.72583912	-5.54606363	h	1	0
31	1.19242644	-5.22209126	-6.74429292	h	1	0
32	-0.9468357	-3.48239107	-4.76817756	h	1	0
33	4.92361423	-11.57377919	-2.62498283	h	1	0
34	6.71295695	-7.22736901	1.98788143	h	1	0
35	4.44174614	-5.01080675	1.00122149	h	1	0
36	10.47609197	-4.12279186	-2.19906905	h	1	0

4*S*_8

Center Number	Atomic Coordinates			Atom	Atomic Number	Atomic Type
	X	Y	Z			
1	-9.32262522	-4.12565552	4.2756891	c	6	0
2	-7.23880525	-6.00709101	3.56956833	c	6	0
3	-7.18564774	-6.61306607	0.73376307	c	6	0
4	-5.15279406	-8.49662127	-0.02464228	c	6	0
5	-2.71042509	-7.34773541	0.47582986	o	8	0
6	-0.67703618	-8.7711735	0.00740733	c	6	0
7	1.75969719	-7.41409658	0.64720446	c	6	0
8	4.05473062	-8.72113953	-0.58603397	c	6	0
9	3.76979539	-8.56339863	-3.49286411	c	6	0
10	4.95560725	-10.45996976	-4.67851364	o	8	0
11	4.85722541	-10.36608026	-7.39463144	c	6	0
12	2.6612424	-6.86213253	-4.56418482	o	8	0
13	4.31698988	-11.22910262	0.27509376	o	8	0
14	6.57675463	-7.41761552	0.15591309	c	6	0
15	6.74398706	-4.67892646	-0.65236673	c	6	0
16	8.17006281	-4.38641529	-2.75475202	o	8	0
17	5.72735776	-2.92111429	0.40815375	o	8	0
18	-0.79739813	-10.93113402	-0.80548988	o	8	0
19	-9.07419026	-2.32532652	3.2818113	h	1	0

20	-11.19694989	-4.85746306	3.78160881	h	1	0
21	-9.31890956	-3.72708076	6.30597935	h	1	0
22	-5.39328811	-5.25405637	4.13378323	h	1	0
23	-7.5001799	-7.76988313	4.63609739	h	1	0
24	-6.92774671	-4.86910667	-0.35919757	h	1	0
25	-9.01260461	-7.40776027	0.14978995	h	1	0
26	-5.24773703	-8.97239233	-2.0350337	h	1	0
27	-5.28453373	-10.25984612	1.05109614	h	1	0
28	1.61470894	-5.43354203	0.09798969	h	1	0
29	2.0023508	-7.46796437	2.70445834	h	1	0
30	2.89921964	-10.42628648	-8.04850878	h	1	0
31	5.89164391	-12.02504468	-8.04258609	h	1	0
32	5.74387196	-8.63434166	-8.08835329	h	1	0
33	2.73163508	-12.06204582	-0.14875863	h	1	0
34	8.11205227	-8.51795825	-0.67419676	h	1	0
35	6.72833229	-7.50122855	2.21475065	h	1	0
36	8.09302125	-2.59995651	-3.1822513	h	1	0

4*S*_9

Center Number	Atomic Coordinates			Atom	Atomic Number	Atomic Type
	X	Y	Z			
1	-7.34004233	-4.62742564	-2.3371168	c	6	0
2	-5.67552847	-4.88901257	0.01758277	c	6	0
3	-5.69610843	-7.57878701	1.10229654	c	6	0
4	-4.15657938	-7.88709951	3.52067723	c	6	0
5	-1.54668449	-7.09840288	3.1724207	o	8	0
6	0.03680494	-8.7674101	2.13738179	c	6	0
7	2.67385371	-7.70362476	1.81574173	c	6	0
8	4.02148653	-8.91118862	-0.47076328	c	6	0
9	2.48575993	-8.32136408	-2.89412188	c	6	0
10	3.23359133	-9.74566014	-4.84760772	o	8	0
11	1.97613407	-9.24558311	-7.20490989	c	6	0

12	0.83356006	-6.73005682	-3.01759122	o	8	0
13	4.33172894	-11.538483	-0.13198285	o	8	0
14	6.74272064	-7.86773663	-0.79932113	c	6	0
15	6.83054024	-5.05134778	-1.28525096	c	6	0
16	6.93988939	-4.51118298	-3.78589514	o	8	0
17	6.76481722	-3.42364952	0.32502477	o	8	0
18	-0.55924221	-10.92699022	1.55928101	o	8	0
19	-6.70274867	-5.89868835	-3.84311343	h	1	0
20	-9.31783028	-5.08783113	-1.92245189	h	1	0
21	-7.28652614	-2.69784101	-3.08279237	h	1	0
22	-3.72701266	-4.3686119	-0.45005146	h	1	0
23	-6.31330212	-3.55292074	1.47459909	h	1	0
24	-4.99433835	-8.91943097	-0.31218241	h	1	0
25	-7.64330683	-8.15265027	1.54570319	h	1	0
26	-4.17643418	-9.84515518	4.17683545	h	1	0
27	-4.84959685	-6.64598751	5.02014478	h	1	0
28	2.58990462	-5.6514603	1.67463337	h	1	0
29	3.76626671	-8.17607688	3.5121321	h	1	0
30	-0.06105456	-9.5226515	-7.01189083	h	1	0
31	2.76907235	-10.58782954	-8.55069777	h	1	0
32	2.33815705	-7.30473844	-7.81241679	h	1	0
33	2.66237279	-12.1786004	0.32013944	h	1	0
34	7.61406304	-8.90258986	-2.35622247	h	1	0
35	7.7732024	-8.26243415	0.94549005	h	1	0
36	6.89365271	-2.67781114	-3.91604	h	1	0

4*S*_10

Center Number	Atomic Coordinates			Atom	Atomic Number	Atomic Type
	X	Y	Z			
1	-10.25304396	-8.65945904	-0.82717186	c	6	0
2	-7.83441347	-8.45767884	0.75129892	c	6	0
3	-6.33237036	-6.0373005	0.20505826	c	6	0

4	-3.91981312	-5.77759271	1.74471008	c	6	0
5	-2.22847983	-7.80631463	1.01454894	o	8	0
6	0.02359723	-7.85594925	2.19999165	c	6	0
7	1.72044907	-9.90697073	1.13925098	c	6	0
8	3.74111795	-8.90303036	-0.73481603	c	6	0
9	2.35562403	-7.57372139	-2.93983619	c	6	0
10	1.79888459	-9.16463863	-4.82898287	o	8	0
11	0.42953164	-8.06209972	-6.90361238	c	6	0
12	1.78079743	-5.35112315	-2.91052406	o	8	0
13	5.07453518	-11.07204814	-1.52311451	o	8	0
14	5.51967919	-6.97523903	0.53700991	c	6	0
15	7.56416357	-6.10366401	-1.25237732	c	6	0
16	8.82847443	-4.08197923	-0.37871155	o	8	0
17	8.08609518	-7.10249384	-3.26476344	o	8	0
18	0.61993114	-6.41085606	3.8793119	o	8	0
19	-11.52427252	-7.06461582	-0.46306898	h	1	0
20	-9.82594368	-8.6655373	-2.85438038	h	1	0
21	-11.28950072	-10.39890557	-0.39994113	h	1	0
22	-8.31395243	-8.51761114	2.77107726	h	1	0
23	-6.6232678	-10.09981375	0.39334294	h	1	0
24	-7.50874183	-4.37505155	0.60866586	h	1	0
25	-5.8524146	-5.93960512	-1.8112122	h	1	0
26	-4.27864609	-5.91450973	3.77874786	h	1	0
27	-2.96427634	-3.97873421	1.3873045	h	1	0
28	2.72609377	-10.80515441	2.70491674	h	1	0
29	0.61079195	-11.33678401	0.14870135	h	1	0
30	1.51996829	-6.52873773	-7.75482291	h	1	0
31	-1.38391321	-7.31562952	-6.25506535	h	1	0
32	0.14683957	-9.59116131	-8.25409312	h	1	0
33	6.32299375	-10.48071754	-2.73530374	h	1	0
34	6.4362411	-7.88207192	2.15859252	h	1	0
35	4.49274636	-5.33978092	1.2587783	h	1	0
36	10.1172595	-3.6864487	-1.62882862	h	1	0

4*S*_11

Center Number	Atomic Coordinates			Atom	Atomic Number	Atomic Type
	X	Y	Z			
1	-7.95433512	-2.98644276	-1.46311001	c	6	0
2	-7.42163531	-5.02749734	0.52119076	c	6	0
3	-4.90104352	-6.39288804	0.07759236	c	6	0
4	-4.41107603	-8.42103825	2.06571185	c	6	0
5	-2.08483074	-9.78017959	1.55840061	o	8	0
6	0.05702377	-8.76570027	2.49408083	c	6	0
7	2.35332761	-10.30248179	1.73218742	c	6	0
8	3.94370192	-9.09078594	-0.41269404	c	6	0
9	2.23113015	-8.80028373	-2.76476163	c	6	0
10	2.23661456	-10.87161625	-4.21905543	o	8	0
11	0.61391595	-10.76351026	-6.39899573	c	6	0
12	0.97563214	-6.92524447	-3.19224	o	8	0
13	5.94584278	-10.80365457	-0.81718257	o	8	0
14	4.94374445	-6.45775535	0.33768469	c	6	0
15	6.60006502	-5.35048057	-1.70495792	c	6	0
16	7.06430681	-2.87890363	-1.35185121	o	8	0
17	7.48474113	-6.52317261	-3.48352866	o	8	0
18	0.13774427	-6.86441775	3.77723702	o	8	0
19	-6.46842165	-1.54386288	-1.46652982	h	1	0
20	-8.0391195	-3.798145	-3.36657932	h	1	0
21	-9.75658129	-2.03609244	-1.10059666	h	1	0
22	-7.41534243	-4.16772684	2.41065123	h	1	0
23	-8.97171148	-6.41087243	0.52228478	h	1	0
24	-3.33983157	-5.03323499	0.10969114	h	1	0
25	-4.883178	-7.26885763	-1.80098359	h	1	0
26	-5.87654534	-9.87874663	2.03670708	h	1	0
27	-4.29951214	-7.59848867	3.95747137	h	1	0
28	3.59018292	-10.48442813	3.37807418	h	1	0
29	1.79549022	-12.18686241	1.10558083	h	1	0
30	0.86955167	-12.56320726	-7.3671467	h	1	0

31	1.15474264	-9.19149572	-7.62394134	h	1	0
32	-1.35433813	-10.51886138	-5.82177936	h	1	0
33	6.94789947	-10.09091939	-2.18350533	h	1	0
34	6.08894647	-6.64418123	2.05420642	h	1	0
35	3.41340776	-5.14728661	0.77107237	h	1	0
36	8.16554349	-2.34888419	-2.72543446	h	1	0

4*S*_12

Center Number	Atomic Coordinates			Atom	Atomic Number	Atomic Type
	X	Y	Z			
1	-8.83966493	-4.20668749	-2.01432864	c	6	0
2	-7.93772923	-5.63704634	0.3360552	c	6	0
3	-5.350779	-6.89609034	-0.03514224	c	6	0
4	-4.48853763	-8.31275854	2.32162861	c	6	0
5	-2.11221982	-9.60879349	1.90602934	o	8	0
6	0.0106944	-8.25560895	2.31122496	c	6	0
7	2.3544777	-9.84569215	1.86569655	c	6	0
8	4.15623821	-8.93093848	-0.28182365	c	6	0
9	2.78194228	-9.19116319	-2.83394265	c	6	0
10	1.02364215	-7.41635843	-3.20737534	o	8	0
11	-0.26703289	-7.5382418	-5.60359558	c	6	0
12	3.19837339	-10.94492609	-4.25637524	o	8	0
13	6.24955353	-10.58826659	-0.32795346	o	8	0
14	5.13895069	-6.24677155	0.2124088	c	6	0
15	6.74730414	-5.27958714	-1.93903733	c	6	0
16	8.4922423	-3.59565214	-1.14055734	o	8	0
17	6.50899164	-5.8516981	-4.14781426	o	8	0
18	0.02408713	-6.06279185	2.99315192	o	8	0
19	-7.5089275	-2.70616459	-2.53173119	h	1	0
20	-9.0189629	-5.47620524	-3.64154786	h	1	0
21	-10.68600554	-3.32780642	-1.69794601	h	1	0
22	-7.83931816	-4.32862264	1.94455739	h	1	0

23	-9.34180459	-7.07836825	0.84829779	h	1	0
24	-3.92857169	-5.4654927	-0.50370799	h	1	0
25	-5.43454454	-8.22575952	-1.6260894	h	1	0
26	-5.8163542	-9.81489447	2.8227256	h	1	0
27	-4.27386683	-7.0230461	3.92158718	h	1	0
28	3.46804185	-9.82822742	3.6095412	h	1	0
29	1.82341759	-11.79898076	1.46438709	h	1	0
30	1.08503938	-7.24552351	-7.13543731	h	1	0
31	-1.65976357	-6.02242846	-5.55939906	h	1	0
32	-1.18555252	-9.37187053	-5.83723316	h	1	0
33	6.01461595	-11.64242228	-1.81825074	h	1	0
34	6.25519607	-6.25685786	1.95060233	h	1	0
35	3.56852223	-4.93508728	0.51242046	h	1	0
36	9.385439	-3.03084876	-2.64440155	h	1	0

6*S*_1

Center Number	Atomic Coordinates			Atom	Atomic Number	Atomic Type
	X	Y	Z			
1	5.26344804	0.71913308	-3.02945553	o	8	0
2	4.83618686	-1.15153562	-1.35485316	c	6	0
3	3.19414767	-0.32074563	0.82419774	c	6	0
4	3.42366361	-2.15119013	3.08620019	c	6	0
5	6.19439182	-2.38627916	4.00683306	c	6	0
6	7.42159251	-0.15287956	3.82550282	o	8	0
7	10.01359771	-0.12200505	4.71640639	c	6	0
8	11.17683359	2.40241936	3.9857379	c	6	0
9	11.44923552	2.80188973	1.12506147	c	6	0
10	12.65307017	5.34929686	0.46079255	c	6	0
11	7.12300619	-4.30232565	4.82691173	o	8	0
12	2.48953419	-4.56113188	2.44952458	o	8	0
13	1.76596265	-1.29464406	5.34701059	c	6	0
14	2.42091848	1.29137544	6.38849184	c	6	0

15	3.63029176	1.11589234	8.62709499	o	8	0
16	4.34867393	3.48678585	9.74497557	c	6	0
17	1.9339562	3.28686894	5.36686537	o	8	0
18	5.69510216	-3.26931816	-1.63945145	o	8	0
19	6.28331167	-0.00861931	-4.37546443	h	1	0
20	3.63049997	1.62640369	1.33764698	h	1	0
21	1.22530395	-0.35191229	0.17542461	h	1	0
22	11.01888303	-1.71867463	3.86861812	h	1	0
23	9.9966191	-0.40242366	6.76678904	h	1	0
24	10.04289733	3.93418632	4.80752888	h	1	0
25	13.04483201	2.50728689	4.88615196	h	1	0
26	12.59676275	1.26153188	0.33542772	h	1	0
27	9.58554426	2.65810003	0.23159632	h	1	0
28	14.54775628	5.52400928	1.2811773	h	1	0
29	12.83207859	5.58635492	-1.58698817	h	1	0
30	11.51437579	6.92507589	1.1760397	h	1	0
31	3.61547922	-5.22195046	1.15811637	h	1	0
32	-0.19142156	-1.24597396	4.68301068	h	1	0
33	1.93343347	-2.73361277	6.81547265	h	1	0
34	2.6850321	4.66270584	10.08493101	h	1	0
35	5.27292682	3.00598159	11.52196514	h	1	0
36	5.64287544	4.50996273	8.50166474	h	1	0

6*S*_2

Center Number	Atomic Coordinates			Atom	Atomic Number	Atomic Type
	X	Y	Z			
1	1.43778762	-1.95229216	-3.46515734	o	8	0
2	3.02478404	-2.64294788	-1.60609388	c	6	0
3	2.22697265	-1.64096247	0.94420475	c	6	0
4	4.3537548	-1.93515201	2.91632514	c	6	0
5	6.62308076	-0.282622	2.09272642	c	6	0
6	8.83870533	-1.26017447	2.82895888	o	8	0

7	11.07925254	0.22622272	2.27485855	c	6	0
8	11.66494278	2.06421733	4.41779196	c	6	0
9	12.26791284	0.77966073	6.94607389	c	6	0
10	12.77932464	2.67637378	9.0730408	c	6	0
11	6.36709497	1.7063379	0.97118478	o	8	0
12	5.01101079	-4.49512486	3.25340641	o	8	0
13	3.49537983	-1.03022234	5.57225878	c	6	0
14	2.73318363	1.72895579	5.65836262	c	6	0
15	4.56632201	3.1755505	6.68496706	o	8	0
16	4.05683575	5.84822423	6.69455868	c	6	0
17	0.74016858	2.56202226	4.88967977	o	8	0
18	4.87103901	-3.96838687	-1.99717725	o	8	0
19	2.07981164	-2.70911874	-5.01288867	h	1	0
20	1.61926821	0.31923879	0.75527497	h	1	0
21	0.58122493	-2.73342741	1.57013567	h	1	0
22	10.78378317	1.22580969	0.49328613	h	1	0
23	12.57965065	-1.17628781	2.04609906	h	1	0
24	13.27638381	3.22925705	3.81696205	h	1	0
25	10.05587265	3.35215703	4.63757298	h	1	0
26	10.68866139	-0.45257776	7.47424593	h	1	0
27	13.91563639	-0.46179037	6.70629442	h	1	0
28	11.12988355	3.88506069	9.4045457	h	1	0
29	13.21879499	1.71384334	10.85145749	h	1	0
30	14.37815011	3.91162394	8.61403412	h	1	0
31	5.60033503	-5.08054607	1.61412346	h	1	0
32	1.86469125	-2.17763258	6.11557494	h	1	0
33	5.04010721	-1.40396692	6.88761903	h	1	0
34	5.67220493	6.71822787	7.63078196	h	1	0
35	3.87604988	6.54456587	4.75864444	h	1	0
36	2.31274574	6.25064729	7.72414883	h	1	0

6*S*_3

Center Number	Atomic Coordinates			Atom	Atomic Number	Atomic Type
	X	Y	Z			
1	3.30568685	-0.54995108	-3.77577959	o	8	0
2	3.95320496	-1.86093416	-1.68665564	c	6	0
3	2.71946186	-0.80996651	0.66104715	c	6	0
4	3.41964399	-2.3476975	3.03486807	c	6	0
5	6.30200386	-2.35715773	3.50668457	c	6	0
6	7.29512299	-0.02230633	3.28503119	o	8	0
7	9.98959646	0.1760271	3.74328816	c	6	0
8	10.77480268	2.89150366	3.23289951	c	6	0
9	13.61661879	3.3030053	3.65078013	c	6	0
10	14.44130013	6.03309987	3.15003422	c	6	0
11	7.49974542	-4.22364506	4.05602438	o	8	0
12	2.56637307	-4.86307451	2.80626143	o	8	0
13	2.06455771	-1.32989241	5.42977336	c	6	0
14	2.59110917	1.41290976	6.06602651	c	6	0
15	3.89148559	1.62607857	8.24970474	o	8	0
16	4.45952779	4.17102788	9.01321569	c	6	0
17	1.91620766	3.21497843	4.81741205	o	8	0
18	5.37079973	-3.66898591	-1.76065136	o	8	0
19	4.16594021	-1.36166847	-5.18280017	h	1	0
20	3.20775433	1.1859977	0.86017609	h	1	0
21	0.66690156	-0.88883639	0.39873183	h	1	0
22	10.96664519	-1.16150813	2.50372352	h	1	0
23	10.3702313	-0.38794914	5.69821488	h	1	0
24	10.26721603	3.38742289	1.28462805	h	1	0
25	9.68949575	4.15780164	4.46692826	h	1	0
26	14.11119756	2.77624636	5.59664016	h	1	0
27	14.68617131	2.01989562	2.41845955	h	1	0
28	13.45284338	7.35574359	4.40046112	h	1	0
29	16.47289376	6.27281147	3.46003877	h	1	0
30	14.03072639	6.59262592	1.19887211	h	1	0
31	3.83051847	-5.75756204	1.82637632	h	1	0
32	0.03216414	-1.52593153	5.09389647	h	1	0

33	2.57998212	-2.55116172	7.00974263	h	1	0
34	2.72165432	5.2559457	9.27615618	h	1	0
35	5.48623973	4.00277192	10.79118148	h	1	0
36	5.61944508	5.11276628	7.58636249	h	1	0

6*S*_4

Center Number	Atomic Coordinates			Atom	Atomic Number	Atomic Type
	X	Y	Z			
1	4.95347135	-1.42577775	-3.19212122	o	8	0
2	3.20674476	-2.38630845	-1.59073095	c	6	0
3	4.41074922	-3.79155462	0.59051372	c	6	0
4	4.48029728	-2.26127321	3.0925835	c	6	0
5	5.98194156	0.20508583	2.61627883	c	6	0
6	8.47387973	-0.12053296	2.89431015	o	8	0
7	10.02875564	2.07341546	2.3815294	c	6	0
8	12.76728078	1.3219235	2.81614996	c	6	0
9	14.59603008	3.52155598	2.33669819	c	6	0
10	17.36513485	2.79044856	2.76308971	c	6	0
11	4.98953191	2.17935073	1.98878686	o	8	0
12	5.69753483	-3.87460928	4.83048668	o	8	0
13	1.80080065	-1.56000925	4.00091076	c	6	0
14	1.88119075	-0.1729106	6.50051811	c	6	0
15	-0.29250055	1.02315361	6.99400588	o	8	0
16	-0.40180428	2.35179716	9.36775429	c	6	0
17	3.68501508	-0.16125216	7.94289313	o	8	0
18	0.95449194	-2.10859263	-1.90511724	o	8	0
19	4.01934362	-0.48360972	-4.46474947	h	1	0
20	3.31186274	-5.50442373	0.94825526	h	1	0
21	6.35101836	-4.32263801	0.13397396	h	1	0
22	9.43743278	3.61284101	3.63264361	h	1	0
23	9.68903321	2.68578636	0.43319871	h	1	0
24	12.97710674	0.63770041	4.76262281	h	1	0

25	13.23537022	-0.2716592	1.57408953	h	1	0
26	14.35439189	4.21038901	0.39293604	h	1	0
27	14.10217244	5.1119863	3.57647493	h	1	0
28	18.62798611	4.39135622	2.41202469	h	1	0
29	17.67983488	2.15429944	4.70892856	h	1	0
30	17.93503595	1.24929845	1.50197345	h	1	0
31	5.73016548	-2.95106456	6.42134737	h	1	0
32	0.78798305	-0.42448481	2.61185537	h	1	0
33	0.72235958	-3.30841742	4.27387834	h	1	0
34	1.03819874	3.83036849	9.43076577	h	1	0
35	-2.28866689	3.17192484	9.45262454	h	1	0
36	-0.09955272	1.04971667	10.9418416	h	1	0

6*S*_5

Center Number	Atomic Coordinates			Atom	Atomic Number	Atomic Type
	X	Y	Z			
1	6.37436761	0.29087932	-2.48803761	o	8	0
2	4.20664244	-0.69169063	-1.54113946	c	6	0
3	4.70483152	-2.83012845	0.29150696	c	6	0
4	4.49229129	-2.05261769	3.11316776	c	6	0
5	6.39261926	0.11775988	3.61216835	c	6	0
6	8.73038166	-0.74694507	4.05112287	o	8	0
7	10.69099226	1.14367605	4.39736179	c	6	0
8	11.84399763	1.93459928	1.8761084	c	6	0
9	13.1666112	-0.21780126	0.4530551	c	6	0
10	14.21548392	0.61502642	-2.11306442	c	6	0
11	5.80245415	2.3368988	3.53604765	o	8	0
12	5.10071921	-4.26733197	4.46482693	o	8	0
13	1.81164193	-1.11548251	3.77161811	c	6	0
14	1.5956149	-0.46118293	6.54708936	c	6	0
15	-0.39261985	1.02736094	7.02364177	o	8	0
16	-0.77172897	1.69887862	9.63462557	c	6	0

17	3.02473979	-1.22668692	8.19224028	o	8	0
18	2.13880487	0.10390394	-2.12006558	o	8	0
19	5.87152568	1.69320261	-3.56555124	h	1	0
20	3.30646184	-4.31291106	-0.04417766	h	1	0
21	6.59222129	-3.61223035	0.00546135	h	1	0
22	12.08278806	0.21562464	5.61100495	h	1	0
23	9.87691235	2.76316425	5.38512526	h	1	0
24	10.35061773	2.75642805	0.69804268	h	1	0
25	13.19912212	3.4601451	2.26351724	h	1	0
26	14.70453879	-0.97220644	1.62767721	h	1	0
27	11.82679171	-1.77385585	0.18178573	h	1	0
28	15.57654303	2.16366895	-1.90650941	h	1	0
29	15.17753539	-0.94374521	-3.07591714	h	1	0
30	12.69451378	1.27623537	-3.35394837	h	1	0
31	4.91946884	-3.82565277	6.24228787	h	1	0
32	1.2573517	0.50739054	2.62970456	h	1	0
33	0.47084318	-2.64385644	3.37083857	h	1	0
34	-2.4626832	2.87368569	9.66040656	h	1	0
35	-1.04030768	0.00626409	10.78686472	h	1	0
36	0.85591751	2.74913114	10.34927148	h	1	0

6S_6

Center Number	Atomic Coordinates			Atom	Atomic Number	Atomic Type
	X	Y	Z			
1	5.32405815	-2.72185871	-3.67225567	o	8	0
2	3.60629715	-1.58170436	-2.16091971	c	6	0
3	2.85563835	-3.23627749	0.04982321	c	6	0
4	3.73394026	-2.27927327	2.68130097	c	6	0
5	6.65360462	-2.35929971	2.78791269	c	6	0
6	7.67713433	-0.38718191	1.53356659	o	8	0
7	10.42087522	-0.28904551	1.43524547	c	6	0
8	11.51413129	1.05956299	3.73559068	c	6	0

9	10.73489988	3.84283511	3.96370097	c	6	0
10	11.83456201	5.12358768	6.31597068	c	6	0
11	7.84684937	-4.0185224	3.80818146	o	8	0
12	2.74592093	-4.06388624	4.39576415	o	8	0
13	2.70188542	0.38358846	3.26456652	c	6	0
14	3.49274418	1.27640806	5.8623508	c	6	0
15	2.63974428	3.62113289	6.34074269	o	8	0
16	3.26331149	4.62311944	8.79215039	c	6	0
17	4.74180911	0.05466869	7.3616986	o	8	0
18	2.77878108	0.51054717	-2.59883411	o	8	0
19	5.68422884	-1.52802681	-5.02297368	h	1	0
20	0.78984686	-3.32345343	0.1042794	h	1	0
21	3.58150834	-5.1510357	-0.19707811	h	1	0
22	10.83207438	0.73490272	-0.31251891	h	1	0
23	11.13863729	-2.21932844	1.29496171	h	1	0
24	10.95897897	0.01553591	5.43705213	h	1	0
25	13.58299718	0.92007155	3.60841953	h	1	0
26	11.34336169	4.86742615	2.26276031	h	1	0
27	8.66654567	3.97256149	4.00362481	h	1	0
28	13.90576316	5.07353477	6.30149895	h	1	0
29	11.25739608	7.10760317	6.43410732	h	1	0
30	11.20238027	4.17491827	8.04575565	h	1	0
31	3.69813108	-3.86932393	5.95045724	h	1	0
32	3.28200676	1.76886926	1.84906722	h	1	0
33	0.63292499	0.31334327	3.20758357	h	1	0
34	2.39160641	6.48765114	8.86487751	h	1	0
35	2.51251284	3.40880072	10.28442331	h	1	0
36	5.31146793	4.76978114	9.0153951	h	1	0

6*S*_7

Center Number	Atomic Coordinates			Atom	Atomic Number	Atomic Type
	X	Y	Z			

1	5.20793499	-1.59716299	-2.9628575	o	8	0
2	3.61454194	-2.76191565	-1.33365413	c	6	0
3	4.98122886	-3.75689096	0.97327716	c	6	0
4	4.81746482	-1.96985648	3.29347087	c	6	0
5	5.959694	0.62228742	2.55113489	c	6	0
6	8.48557919	0.54836228	2.47513148	o	8	0
7	9.73805111	2.85659266	1.69333874	c	6	0
8	12.56980566	2.37215589	1.73391827	c	6	0
9	13.62498422	1.77298592	4.36860281	c	6	0
10	16.47783139	1.28252419	4.34868735	c	6	0
11	4.69664571	2.47037812	2.03855954	o	8	0
12	6.25495311	-3.1832248	5.18595963	o	8	0
13	2.06095251	-1.57184199	4.1529017	c	6	0
14	1.92180822	0.07627376	6.48614195	c	6	0
15	-0.34866389	1.15336859	6.75958362	o	8	0
16	-0.66163759	2.71553663	8.96745763	c	6	0
17	3.64620544	0.36155494	7.99682927	o	8	0
18	1.36152117	-2.92466415	-1.71357053	o	8	0
19	4.16004014	-0.93949325	-4.32292208	h	1	0
20	4.12556815	-5.55655317	1.51505155	h	1	0
21	6.98123616	-4.05099663	0.56399071	h	1	0
22	9.18996783	4.37392396	2.99061178	h	1	0
23	9.06105961	3.36188403	-0.19485176	h	1	0
24	13.00242874	0.81792565	0.42936409	h	1	0
25	13.50168367	4.06155585	0.96644619	h	1	0
26	13.19811332	3.35310221	5.64692131	h	1	0
27	12.63124345	0.12287376	5.12952014	h	1	0
28	17.18218711	0.86322151	6.2486812	h	1	0
29	16.95127745	-0.3280918	3.13490685	h	1	0
30	17.52192482	2.92633874	3.63948962	h	1	0
31	6.04686375	-2.151583	6.69784251	h	1	0
32	0.89276864	-0.76339977	2.66117535	h	1	0
33	1.26181706	-3.42241939	4.63505231	h	1	0
34	0.65744547	4.30323433	8.91467904	h	1	0
35	-2.60733908	3.38574781	8.89532226	h	1	0

36	-0.32922674	1.61851445	10.68523229	h	1	0

6*S*_8

Center Number	Atomic Coordinates			Atom	Atomic Number	Atomic Type
	X	Y	Z			
1	4.68628255	-2.33493571	-3.47098816	o	8	0
2	2.80040183	-2.51708081	-1.75472315	c	6	0
3	3.58888701	-4.01895665	0.54895732	c	6	0
4	4.20611359	-2.36366633	2.89166145	c	6	0
5	6.37935589	-0.53806988	2.17610737	c	6	0
6	8.65605413	-1.59034519	2.51406257	o	8	0
7	10.82701645	-0.05482306	1.84995906	c	6	0
8	11.66725764	1.57362173	4.07328636	c	6	0
9	14.05252854	3.10725565	3.4613283	c	6	0
10	14.93703778	4.74012779	5.68307828	c	6	0
11	6.0143649	1.56810377	1.33490203	o	8	0
12	4.92051653	-4.10821143	4.77566318	o	8	0
13	1.90524348	-0.78662203	3.73600368	c	6	0
14	2.49551383	0.73577233	6.08343032	c	6	0
15	0.78036058	2.5503734	6.49307439	o	8	0
16	1.16139075	4.06212005	8.72454418	c	6	0
17	4.29244078	0.35581981	7.48421123	o	8	0
18	0.74304673	-1.55947927	-2.0702937	o	8	0
19	4.04357433	-1.27497497	-4.82857067	h	1	0
20	2.03123105	-5.2693933	1.07589048	h	1	0
21	5.25625035	-5.16191338	0.13885372	h	1	0
22	10.34740563	1.10704773	0.21008055	h	1	0
23	12.28369432	-1.42254366	1.32454686	h	1	0
24	10.11880173	2.85063335	4.58565977	h	1	0
25	12.01186329	0.34888905	5.71073594	h	1	0
26	15.58039715	1.81654068	2.9028888	h	1	0
27	13.68822272	4.3183881	1.8154283	h	1	0

28	15.38049094	3.57530764	7.33741751	h	1	0
29	16.6360591	5.81677192	5.19636932	h	1	0
30	13.47260567	6.0924241	6.24453645	h	1	0
31	5.32180935	-3.09665074	6.25807217	h	1	0
32	1.2442638	0.47106869	2.24400747	h	1	0
33	0.35091335	-2.08044435	4.18573419	h	1	0
34	-0.41286713	5.38908677	8.76553748	h	1	0
35	1.15922172	2.87488487	10.41426759	h	1	0
36	2.95973519	5.07021016	8.61158155	h	1	0

6*S*_9

Center Number	Atomic Coordinates			Atom	Atomic Number	Atomic Type
	X	Y	Z			
1	5.05638923	-0.81910478	-3.20394753	o	8	0
2	3.48123057	-2.06054241	-1.61835594	c	6	0
3	4.90274793	-3.40190874	0.47079976	c	6	0
4	4.7931177	-2.02436548	3.05938123	c	6	0
5	5.92948904	0.65069471	2.71816072	c	6	0
6	8.43852214	0.67682653	3.02725917	o	8	0
7	9.66715471	3.09612314	2.63846255	c	6	0
8	12.48033009	2.7409837	3.10383531	c	6	0
9	13.76759445	0.93100331	1.23882083	c	6	0
10	16.60227867	0.62990921	1.74342249	c	6	0
11	4.6686322	2.48729454	2.16001439	o	8	0
12	6.24711469	-3.54900847	4.69180713	o	8	0
13	2.05403089	-1.75726397	4.02275651	c	6	0
14	1.97515146	-0.51889462	6.5990729	c	6	0
15	-0.33936303	0.32387412	7.17648363	o	8	0
16	-0.60380569	1.482811	9.62558612	c	6	0
17	3.78095994	-0.3308548	8.02649777	o	8	0
18	1.2052768	-2.05724889	-1.88046633	o	8	0
19	3.98248629	0.0496672	-4.4171627	h	1	0

20	4.06184271	-5.27058972	0.73540164	h	1	0
21	6.89098318	-3.62798035	-0.03016429	h	1	0
22	8.83280687	4.47287733	3.93699433	h	1	0
23	9.27116081	3.72890245	0.70709287	h	1	0
24	13.36881966	4.61474274	2.99440994	h	1	0
25	12.75925882	2.06998531	5.04701042	h	1	0
26	12.83553062	-0.91563792	1.34290334	h	1	0
27	13.47353904	1.6259484	-0.69617479	h	1	0
28	16.94831882	-0.12066763	3.64301404	h	1	0
29	17.4736237	-0.66892138	0.38854872	h	1	0
30	17.59132817	2.44491802	1.60008117	h	1	0
31	6.18030238	-2.71811756	6.33180625	h	1	0
32	0.87619891	-0.69564526	2.70756168	h	1	0
33	1.23198803	-3.65119065	4.19776709	h	1	0
34	-2.58823551	2.01337979	9.77134796	h	1	0
35	-0.0929495	0.1495811	11.11762324	h	1	0
36	0.60727151	3.14897143	9.76662206	h	1	0

6S_10

Center Number	Atomic Coordinates			Atom	Atomic Number	Atomic Type
	X	Y	Z			
1	5.96876721	-0.4155787	-2.76995367	o	8	0
2	3.80744365	-1.15124399	-1.61447494	c	6	0
3	4.26780225	-3.20367014	0.32432814	c	6	0
4	4.23915152	-2.25030242	3.10003091	c	6	0
5	6.32714924	-0.21902233	3.37332026	c	6	0
6	8.57651158	-1.23289982	3.93661206	o	8	0
7	10.70157514	0.49255353	4.06786235	c	6	0
8	11.96808659	0.76419243	1.4936831	c	6	0
9	14.26300388	2.53590744	1.59027788	c	6	0
10	15.57656719	2.81264377	-0.97539308	c	6	0
11	5.93899362	2.018956	3.02711528	o	8	0

12	4.72676578	-4.42494634	4.55965487	o	8	0
13	1.67503045	-1.05698085	3.8054284	c	6	0
14	1.63295625	-0.23276351	6.54323848	c	6	0
15	-0.25726951	1.37985497	7.02126642	o	8	0
16	-0.47256642	2.223085	9.6007609	c	6	0
17	3.10942635	-0.96479048	8.16100098	o	8	0
18	1.76885784	-0.23186306	-2.11065396	o	8	0
19	5.50068979	0.95084543	-3.90726617	h	1	0
20	2.76830832	-4.61412175	0.14577244	h	1	0
21	6.0881237	-4.11895478	0.00086304	h	1	0
22	11.97764367	-0.35755253	5.45300681	h	1	0
23	10.03671575	2.31728592	4.77092927	h	1	0
24	12.54829044	-1.11237161	0.82743179	h	1	0
25	10.5746748	1.4795481	0.13738114	h	1	0
26	13.66044495	4.4046449	2.26381154	h	1	0
27	15.62487925	1.82939058	2.98973228	h	1	0
28	17.20065659	4.08932272	-0.85589754	h	1	0
29	16.26154157	0.98434055	-1.6667645	h	1	0
30	14.27987876	3.57337417	-2.39999408	h	1	0
31	4.70359106	-3.85945488	6.31008611	h	1	0
32	1.2098943	0.54269738	2.5931826	h	1	0
33	0.19335885	-2.48151569	3.54265546	h	1	0
34	1.24199562	3.21852682	10.17734478	h	1	0
35	-2.09429007	3.4917161	9.63122506	h	1	0
36	-0.77760577	0.61880271	10.86500072	h	1	0

6*S*_11

Center Number	Atomic Coordinates			Atom	Atomic Number	Atomic Type
	X	Y	Z			
1	4.85195626	0.97032978	-2.99926088	o	8	0
2	4.88858173	-0.86613001	-1.23418807	c	6	0
3	3.11759243	-0.32462834	0.93509101	c	6	0

4	3.59087161	-2.12651013	3.17961601	c	6	0
5	6.34811345	-1.93313475	4.14183386	c	6	0
6	7.12881551	0.49978195	4.21762161	o	8	0
7	9.68715712	0.93688121	5.11668484	c	6	0
8	11.60174919	0.77605649	2.9681866	c	6	0
9	11.26585107	2.79883765	0.91737306	c	6	0
10	13.19407491	2.54334724	-1.22751751	c	6	0
11	7.60967471	-3.72402162	4.78365014	o	8	0
12	3.06915165	-4.64510771	2.49228946	o	8	0
13	1.7682279	-1.58597739	5.41101547	c	6	0
14	1.93900542	1.06158692	6.49139032	c	6	0
15	2.9913589	1.05172156	8.8137075	o	8	0
16	3.25493967	3.49349366	9.97696745	c	6	0
17	1.21660798	2.96632255	5.43659376	o	8	0
18	6.21984439	-2.73372199	-1.43623407	o	8	0
19	6.00335242	0.43793691	-4.33008034	h	1	0
20	3.25784962	1.65952134	1.47731515	h	1	0
21	1.18111545	-0.62597961	0.2596322	h	1	0
22	10.10652737	-0.44580302	6.59068522	h	1	0
23	9.62586744	2.83333002	5.93672198	h	1	0
24	13.49313955	0.94485939	3.81087611	h	1	0
25	11.5002504	-1.11900599	2.13597923	h	1	0
26	9.34679561	2.6874197	0.14283347	h	1	0
27	11.42811158	4.68107831	1.78013844	h	1	0
28	13.02650489	0.70457402	-2.16702267	h	1	0
29	12.92662724	4.01286489	-2.66010787	h	1	0
30	15.13428334	2.70409591	-0.51894681	h	1	0
31	4.39147473	-5.14357643	1.32197344	h	1	0
32	-0.15421607	-1.86679744	4.70144231	h	1	0
33	2.14009169	-2.99234942	6.87323164	h	1	0
34	4.1065669	3.14248249	11.81957372	h	1	0
35	4.46843028	4.71118382	8.83158867	h	1	0
36	1.41160677	4.40081515	10.18792141	h	1	0

6S_12

Center Number	Atomic Coordinates			Atom	Atomic Number	Atomic Type
	X	Y	Z			
1	4.27905961	-0.51221766	-3.80226719	o	8	0
2	4.33329988	-1.94570572	-1.69816177	c	6	0
3	3.1542631	-0.62929771	0.54037433	c	6	0
4	3.48117108	-2.17474972	2.98947834	c	6	0
5	6.30466809	-2.61603473	3.6269067	c	6	0
6	7.69943814	-0.53167256	3.16332179	o	8	0
7	10.37521707	-0.71355864	3.73405997	c	6	0
8	11.65709767	1.65837374	2.73891781	c	6	0
9	10.78431921	4.11485433	4.01116216	c	6	0
10	12.12925717	6.45603992	2.96527271	c	6	0
11	7.1396103	-4.56217114	4.48056392	o	8	0
12	2.2481227	-4.52916706	2.78523953	o	8	0
13	2.18942751	-0.88771843	5.28109694	c	6	0
14	3.15158624	1.7264507	5.95728709	c	6	0
15	3.94038925	1.80885517	8.37904204	o	8	0
16	4.80060787	4.23735577	9.24827094	c	6	0
17	3.17139566	3.54807273	4.56024644	o	8	0
18	5.2299044	-4.06608567	-1.68117681	o	8	0
19	5.03281389	-1.52341237	-5.13974455	h	1	0
20	3.91836616	1.27806585	0.71802268	h	1	0
21	1.12742833	-0.4268818	0.1574365	h	1	0
22	11.11787253	-2.42794092	2.8503999	h	1	0
23	10.5946033	-0.91295673	5.78425824	h	1	0
24	13.70190143	1.41633517	3.00805432	h	1	0
25	11.34520752	1.7766325	0.69093086	h	1	0
26	8.73651538	4.31613084	3.7702766	h	1	0
27	11.12909336	3.97255457	6.0557327	h	1	0
28	11.77366326	6.67767204	0.93664863	h	1	0
29	11.47842007	8.18088671	3.90473269	h	1	0
30	14.18054365	6.32990264	3.23027096	h	1	0

31	3.24857223	-5.53377727	1.62174794	h	1	0
32	0.17640048	-0.72238452	4.82283248	h	1	0
33	2.37021277	-2.15551348	6.897177	h	1	0
34	3.33364891	5.66586956	8.98021823	h	1	0
35	5.22209398	3.99115643	11.24939787	h	1	0
36	6.49406089	4.81599965	8.21721342	h	1	0

6S_13

Center Number	Atomic Coordinates			Atom	Atomic Number	Atomic Type
	X	Y	Z			
1	6.14763572	-1.59604692	-3.09914369	o	8	0
2	4.10159424	-0.84054538	-1.76288914	c	6	0
3	3.13622812	-2.89296454	-0.01944455	c	6	0
4	3.51841492	-2.37915518	2.84501817	c	6	0
5	6.38717468	-2.346659	3.41177657	c	6	0
6	7.44514464	-0.14238936	2.68380688	o	8	0
7	10.16259683	0.03232269	2.97204961	c	6	0
8	10.9894126	2.59389363	1.96812132	c	6	0
9	13.85745568	2.97535802	2.16995426	c	6	0
10	14.72143496	5.5495697	1.16396175	c	6	0
11	7.54335525	-4.09147885	4.32291205	o	8	0
12	2.40498183	-4.47932276	4.04243158	o	8	0
13	2.22459797	0.09708741	3.68239224	c	6	0
14	2.46911943	0.51794311	6.49974101	c	6	0
15	1.51926733	2.76795033	7.19398197	o	8	0
16	1.61829204	3.31523576	9.85763855	c	6	0
17	3.39002535	-0.97798812	7.98929549	o	8	0
18	3.17731675	1.24257983	-2.00503123	o	8	0
19	6.61637018	-0.16819373	-4.15775219	h	1	0
20	1.09948596	-3.07958776	-0.32560143	h	1	0
21	4.030297	-4.69920663	-0.45899087	h	1	0
22	11.03879386	-1.52807328	1.93259008	h	1	0

23	10.63148903	-0.20827404	4.97348163	h	1	0
24	10.39066981	2.766111	-0.01065887	h	1	0
25	10.00082965	4.08564685	3.01626868	h	1	0
26	14.44114854	2.77598117	4.15211809	h	1	0
27	14.82895452	1.464239	1.12976056	h	1	0
28	13.83741495	7.09942325	2.21569359	h	1	0
29	16.77249481	5.76840509	1.32484831	h	1	0
30	14.21768711	5.78226371	-0.83179507	h	1	0
31	2.95307261	-4.41733895	5.79253788	h	1	0
32	2.95555277	1.73848672	2.66917714	h	1	0
33	0.20755678	-0.02948752	3.22763401	h	1	0
34	3.57477402	3.34698156	10.51825902	h	1	0
35	0.74946275	5.17011248	10.07289867	h	1	0
36	0.57375053	1.8924075	10.92989872	h	1	0

6*S*_14

Center Number	Atomic Coordinates			Atom	Atomic Number	Atomic Type
	X	Y	Z			
1	4.97530916	1.17256509	-2.95119533	o	8	0
2	4.84503399	-0.75800459	-1.29592602	c	6	0
3	3.1286921	-0.19299214	0.91044873	c	6	0
4	3.54348744	-2.08387533	3.0922161	c	6	0
5	6.32903514	-2.10011197	3.99424377	c	6	0
6	7.35264735	0.23799857	3.89111927	o	8	0
7	9.95881511	0.45085638	4.71423242	c	6	0
8	10.95601093	3.0204744	3.89276104	c	6	0
9	10.9662197	3.48597527	1.02242593	c	6	0
10	12.5558042	1.59567362	-0.49582667	c	6	0
11	7.42982591	-3.95797587	4.73170298	o	8	0
12	2.83819637	-4.54308612	2.35295058	o	8	0
13	1.8228575	-1.48412387	5.38812947	c	6	0
14	2.2391055	1.10698296	6.53436076	c	6	0

15	3.5098322	0.95760095	8.74043348	o	8	0
16	4.025628	3.33901747	9.94411215	c	6	0
17	1.53563852	3.08545183	5.61052576	o	8	0
18	5.998904	-2.72577854	-1.6141094	o	8	0
19	6.07714205	0.61788072	-4.31465723	h	1	0
20	3.36954559	1.7655687	1.50595535	h	1	0
21	1.17160894	-0.38784586	0.25486089	h	1	0
22	11.03105972	-1.11528021	3.89983193	h	1	0
23	10.00817143	0.23921157	6.7728129	h	1	0
24	9.84472761	4.5018723	4.82547832	h	1	0
25	12.89031965	3.1812018	4.63201427	h	1	0
26	9.01713669	3.47037677	0.31988224	h	1	0
27	11.6811227	5.4037887	0.69128239	h	1	0
28	11.75875667	-0.31347544	-0.4312599	h	1	0
29	12.65397217	2.15216051	-2.48714099	h	1	0
30	14.49933291	1.49486152	0.21649969	h	1	0
31	4.05416252	-5.06525254	1.0812892	h	1	0
32	-0.13401685	-1.59126452	4.72944747	h	1	0
33	2.13387813	-2.96053164	6.79451803	h	1	0
34	5.02330528	2.87692499	11.68599977	h	1	0
35	5.19656426	4.52590135	8.72427149	h	1	0
36	2.26676204	4.34015024	10.35642075	h	1	0

6*S*_15

Center Number	Atomic Coordinates			Atom	Atomic Number	Atomic Type
	X	Y	Z			
1	1.478787	-1.75228495	-3.48573359	o	8	0
2	2.97005126	-2.56684582	-1.59712703	c	6	0
3	2.07597347	-1.67951803	0.96350011	c	6	0
4	4.08727595	-2.15938182	3.018508	c	6	0
5	6.44644954	-0.54131654	2.4111038	c	6	0
6	8.59501091	-1.67286447	3.1233624	o	8	0

7	10.90712691	-0.24051704	2.78742842	c	6	0
8	11.49038918	1.33100511	5.13201985	c	6	0
9	13.97508838	2.80380914	4.8679049	c	6	0
10	14.59962335	4.3829717	7.21365436	c	6	0
11	6.30606986	1.5483383	1.46550242	o	8	0
12	4.64302163	-4.75607576	3.23657052	o	8	0
13	3.12612063	-1.37795334	5.6782892	c	6	0
14	2.41643943	1.38927128	5.88185142	c	6	0
15	4.21837497	2.73058823	7.08842978	o	8	0
16	3.76996337	5.41058627	7.21783751	c	6	0
17	0.48495068	2.31024324	5.05743885	o	8	0
18	4.8108298	-3.90308369	-1.97602925	o	8	0
19	2.17765767	-2.44009624	-5.04097914	h	1	0
20	1.54662724	0.30951649	0.85629404	h	1	0
21	0.36197916	-2.74088513	1.44283627	h	1	0
22	10.71560469	0.95406811	1.1133177	h	1	0
23	12.36153157	-1.66930083	2.45361919	h	1	0
24	9.91933801	2.64123318	5.4617212	h	1	0
25	11.58324629	0.07366596	6.77913009	h	1	0
26	15.52871458	1.47755526	4.49553599	h	1	0
27	13.86536459	4.04504927	3.20764423	h	1	0
28	14.78481591	3.18663778	8.89437446	h	1	0
29	16.37803228	5.41369445	6.97511489	h	1	0
30	13.11216205	5.77255201	7.59561084	h	1	0
31	5.31979259	-5.2624691	1.60512288	h	1	0
32	1.4487238	-2.51569715	6.08203028	h	1	0
33	4.6004726	-1.8566977	7.03964304	h	1	0
34	5.34493781	6.18554099	8.29584077	h	1	0
35	3.72364943	6.21557644	5.31648956	h	1	0
36	1.97607578	5.80231333	8.16255766	h	1	0

6*S*_16

Center Number	Atomic Coordinates			Atom	Atomic Number	Atomic Type
	X	Y	Z			
1	1.22149725	-1.53885569	-3.23725669	o	8	0
2	2.98842446	-2.16412976	-1.52306152	c	6	0
3	2.02852891	-1.98083033	1.1579837	c	6	0
4	4.21111376	-2.14549072	3.08408827	c	6	0
5	5.95893366	0.17045018	2.70904043	c	6	0
6	8.3672441	-0.33548481	3.28665551	o	8	0
7	10.11049732	1.76561993	3.08002375	c	6	0
8	12.71901931	0.80879316	3.81078754	c	6	0
9	14.73196412	2.89206852	3.66042043	c	6	0
10	17.37219586	1.95494202	4.39282188	c	6	0
11	5.185795	2.22686067	2.03724966	o	8	0
12	5.51474869	-4.46030815	2.86946809	o	8	0
13	3.22110407	-2.1004259	5.84494522	c	6	0
14	1.79320422	0.29109678	6.51056907	c	6	0
15	3.24045479	1.88582364	7.87422002	o	8	0
16	2.10346925	4.29282904	8.43029625	c	6	0
17	-0.36474258	0.74904943	5.88452815	o	8	0
18	5.10883663	-2.83447379	-2.13510972	o	8	0
19	1.99717674	-1.7462778	-4.89123596	h	1	0
20	0.9363407	-0.24688481	1.38120334	h	1	0
21	0.73282882	-3.56493518	1.4799271	h	1	0
22	9.46278163	3.28854767	4.32406983	h	1	0
23	10.06004872	2.4810551	1.13853016	h	1	0
24	12.64049542	0.03721998	5.73410286	h	1	0
25	13.23396333	-0.76460426	2.56164886	h	1	0
26	14.78247596	3.66543893	1.73452237	h	1	0
27	14.18922999	4.46754589	4.89925955	h	1	0
28	17.99427926	0.4219356	3.14669376	h	1	0
29	18.77038974	3.47503901	4.26765351	h	1	0
30	17.39808864	1.23210737	6.33351389	h	1	0
31	6.18997891	-4.49713086	1.1609002	h	1	0
32	1.94911975	-3.71289632	6.07097041	h	1	0

33	4.84609064	-2.3502943	7.09147239	h	1	0
34	3.48225481	5.30633083	9.5771636	h	1	0
35	1.72049064	5.31841319	6.67912546	h	1	0
36	0.33606905	4.04022338	9.46785875	h	1	0
